# A Second-Site Noncomplementation Screen for Modifiers of Rho1 Signaling during Imaginal Disc Morphogenesis in *Drosophila*

**DOI:** 10.1371/journal.pone.0007574

**Published:** 2009-10-23

**Authors:** Kistie Patch, Shannon R. Stewart, Aaron Welch, Robert E. Ward

**Affiliations:** Department of Molecular Biosciences, University of Kansas, Lawrence, Kansas, United States of America; University of Oldenburg, Germany

## Abstract

**Background:**

Rho1 is a small GTPase of the Ras superfamily that serves as the central component in a highly conserved signaling pathway that regulates tissue morphogenesis during development in all animals. Since there is tremendous diversity in the upstream signals that can activate Rho1 as well as the effector molecules that carry out its functions, it is important to define relevant *Rho1*-interacting genes for each morphogenetic event regulated by this signaling pathway. Previous work from our lab and others has shown that Rho signaling is necessary for the morphogenesis of leg imaginal discs during metamorphosis in *Drosophila*, although a comprehensive identification of *Rho1*-interacting genes has not been attempted for this process.

**Methodology/Principal Findings:**

We characterized an amorphic allele of *Rho1* that displays a poorly penetrant dominant malformed leg phenotype and is capable of being strongly enhanced by *Rho1*-interacting heterozygous mutations. We then used this allele in a second-site noncomplementation screen with the Exelixis collection of molecularly defined deficiencies to identify *Rho1*-interacting genes necessary for leg morphogenesis. In a primary screen of 461 deficiencies collectively uncovering ∼50% of the *Drosophila* genome, we identified twelve intervals harboring *Rho1*-interacting genes. Through secondary screening we identified six *Rho1*-interacting genes including three that were previously identified (*RhoGEF2*, *broad*, and *stubbloid*), thereby validating the screen. In addition, we identified *Cdc42*, *Rheb* and *Sc2* as novel *Rho1*-interacting genes involved in adult leg development.

**Conclusions/Significance:**

This screen identified well-known and novel *Rho1*-interacting genes necessary for leg morphogenesis, thereby increasing our knowledge of this important signaling pathway. We additionally found that Rheb may have a unique function in leg morphogenesis that is independent of its regulation of Tor.

## Introduction

Cell shape changes, cell rearrangements, oriented cell divisions and regulated cell death collectively shape tissues and ultimately influence the development of the organism as a whole. These processes, generically referred to as morphogenesis, are the underlying mechanisms for numerous developmental events including gastrulation, neurulation, organogenesis and metamorphosis. Morphogenetic processes are largely driven by regulated changes of the actin cytoskeleton. Members of the highly conserved Rho family of small GTPases are key regulators of the actin cytoskeleton (reviewed in [1], [2]), and genetic and pharmacological studies in a variety of organisms have demonstrated critical roles for Rho, Rac and Cdc42 in regulating specific morphogenetic events during development. For example, Rho signaling has been implicated in neural tube closure [3] and cardiac morphogenesis [4] during vertebrate embryogenesis, whereas Rac signaling is critical for neurite outgrowth in vertebrates, worms and flies [5]-[7], and Cdc42 signaling plays similar roles in neurite outgrowth and axon guidance in vertebrates [8], [9].

*Drosophila* melanogaster has proven to be an excellent model organism for elucidating the function of Rho proteins during development and for identifying genes that function in concert with Rho proteins to transduce signals through these GTPases (reviewed in [10]). The *Drosophila* genome encodes one Rho gene (*Rho1*), one Cdc42 gene (*Cdc42*), three Rac genes (*Rac1*, *Rac2*, and *Mtl*), and several additional, more divergent, Rho family genes (e.g. *RhoBTB* and *RhoL*). In *Drosophila*, loss of function genetic analysis has demonstrated a role for *Rho1* signaling during oogenesis, and for cellularization of the blastoderm embryo, gastrulation, dorsal closure, and head involution during embryogenesis [11]–[13]. Similarly, complete loss of all three *Rac* genes leads to defects in dorsal closure and neural development [7], whereas zygotic loss of *Cdc42* results in defects in germ band retraction during embryogenesis [14].

Like all members of the Rho family, Rho1 functions primarily as a molecular switch, alternating between an inactive, GDP-bound state and an active, GTP-bound form (reviewed in [1]). Guanine nucleotide exchange factors (GEFs) activate Rho1 by removing bound GDP, whereas GTPase activating proteins (GAPs) stimulate the weak GTPase activity of Rho1, thereby inactivating it. In certain contexts, guanine nucleotide dissociation inhibitors (GDIs) bind to and sequester GDP-bound Rho1, reinforcing the inactive Rho1 state. A number of effector proteins can bind activated Rho1 to transduce signals in specific ways. For example, Rho kinase is a serine/threonine kinase that regulates contractile events at the actin cytoskeleton primarily by phosphorylating and thereby inactivating the myosin binding subunit of the myosin phosphatase complex (reviewed in [15]). Since myosin phosphatase normally dephosphorylates the myosin regulatory light chain (MRLC; encoded by *spaghetti squash* or *sqh* in *Drosophila*), the net effect of activated Rho1 signaling through Rho kinase is an increase in the phosphorylation of MRLC. Phospho-MRLC induces a conformational change in the myosin heavy chain (encoded by *zipper* or *zip* in *Drosophila*) that increases the ability of myosin to bind to actin filaments, thereby generating a chemomechanical force on the actin cytoskeleton sufficient for cell shape changes.

Less is known about the upstream events that activate Rho1 signaling during *Drosophila* development. RhoGEF proteins directly activate Rho1, but the *Drosophila* genome encodes more than 20 genes that are predicted to have RhoGEF activity, some of which may have preferential specificity for a single Rho family member, whereas others may be more promiscuous. In addition, the mechanisms by which specific RhoGEFs are activated to signal through Rho1 are complex and not fully understood.

The morphogenesis of leg imaginal discs that occurs during *Drosophila* metamorphosis is a particularly useful genetic model for studying Rho1 signaling. Adult legs are derived from imaginal discs that were specified during embryogenesis and underwent extensive proliferation and patterning during larval development (reviewed in [16]). At the end of the third larval instar each of the leg imaginal discs consists of a single-layered columnar epithelium that is covered and apposed by a squamous peripodial epithelium. In response to the late larval pulse of the steroid hormone ecdysone that triggers puparium formation and initiates metamorphosis, these flat epithelial discs are transformed into rudimentary adult legs in approximately 12 hours (reviewed in [17]). Classical studies by the Fristrom lab and more recent imaging studies have revealed that this morphogenetic process is largely driven by changes in cell shape and by cell rearrangements [18]–[20]. Furthermore, studies by Fristrom and Fristrom [21] demonstrated that the elongation and eversion of the leg imaginal discs could be reversibly inhibited by cytochalasin B, indicating a central role for the actin cytoskeleton in driving leg disc morphogenesis. Not surprisingly, independent genetic modifier screens using *zip* and an ecdysone-induced transcription factor, *broad*, have identified genes in the Rho signaling pathway as playing a critical role in regulating leg morphogenesis [22]–[25]. Similar experiments also demonstrated robust genetic interactions between the ecdysone-induced type II transmembrane serine protease *stubbloid* (*sbd*) and components of the Rho signaling pathway [26].

As a means to identify genes that function in concert with *Rho1* during leg morphogenesis we have conducted a modifier screen using an amorphic allele of *Rho1* and the Exelixis collection of molecularly defined deficiencies [27]. Screening through a collection of 461 deficiencies that collectively uncover ∼50% of the *Drosophila* genome, we identified 12 deficiencies that likely contain *Rho1*-interacting genes necessary for leg morphogenesis. Included in this set were deficiencies that removed *broad*, *RhoGEF2* and *stubbloid*, three genes that had previously been identified as interacting with *Rho1* during leg imaginal disc morphogenesis. Further, we were able to identify *Cdc42*, *Rheb*, and *Sc2* as likely *Rho1*-interacting genes.

## Materials and Methods

### *Drosophila* stocks

All *Drosophila* stocks were maintained on media consisting of corn meal, sugar, yeast, and agar in incubators maintained at a constant temperature of 21°C, or in a room that typically fluctuated between 21°C and 22.5°C. Many of the deficiency stocks used in this study were generated by Exelixis, Inc., and obtained from the Bloomington *Drosophila* stock center at Indiana University (Bloomington, IN) [27]. Other deficiency stocks and specific mutations used in the screen were also obtained from the Bloomington *Drosophila* stock center. The *Rho1^E(br)233^* and *Rho1^E(br)246^* stocks used in this study were isolated in a screen for dominant modifiers of *broad* [25]. The *zip^E(br)^*, *Rho1^J3.8^*, and *Rho1^E3.10^* stocks were obtained from S. Halsell (James Madison University; [23], [28]). The *RhoGEF2^11-3^* stock was obtained from L. von Kalm (University of Central Florida; [26]). *Dll-Gal4*, *UAS-Rheb.Pa2*, *UAS-Rheb.Pa3*, and *UAS-PI3K92E.CAAX* were obtained from the Bloomington *Drosophila* stock center. Genetic experiments were conducted in incubators controlled at a constant temperature of either 21°C or 25°C, as indicated.

### Characterization of *Rho1^E(br)233^* and *Rho1^E(br)246^*

*Rho1^E(br)233^* and *Rho1^E(br)246^* were balanced with *CyO, P{w^+^, Dfd-EYFP}* [29] to allow for unambiguous identification of homozygous mutant embryos starting at ∼12 hours after egg laying. Genomic DNA isolated from homozygous mutant late embryos was sequenced at the DNA Facility of the Iowa State University Office of Biotechnology (Ames, IA). Lethal phase analyses were performed by collecting homozygous mutant embryos produced through a four hour egg lay of *Rho1^E(br)246^/CyO, P{w^+^, ActGFP}* or *Rho1^E(br)233^/CyO, P{w^+^, ActGFP}* at 25°C, and determining the percentage of unhatched embryos after 48 hours. Non-hatched embryos were then dechorionated in 50% bleach, mounted on microscope slides in Hoyer's medium and subsequently examined for cuticular phenotypes on a Nikon Eclipse 80*i* compound microscope.

### RNA isolation and northern blot analysis

Non YFP-expressing embryos were isolated from 4 hour collections of *Rho1^E(br)246^/CyO, P{w^+^, Dfd-EYFP}*, *Rho1^E(br)233^/CyO, P{w^+^, Dfd-EYFP}*, or *w^1118^* that were aged to be 12–16 hours after egg laying (AEL), 16–20 AEL, or 20–24 hours AEL. ∼100 embryos from each collection were dechorionated and lysed in Tripure isolation reagent (Roche Applied Science, Indianapolis, IN). Total RNA was extracted from these lysates, and approximately 10 µg of total RNA per sample were separated by formaldehyde agarose gel electrophoresis and transferred to a nylon membrane (GeneScreen Plus, PerkinElmer, Waltham, MA). The membrane was hybridized and stripped as described by [30]. Specific probes were labeled by random priming of gel-purified fragments (Stratagene, La Jolla, CA). Generation of probe fragments for *Rho1* is described in [25], and for *rp49* in [31].

### Protein isolation and western blot analysis

Non YFP-expressing embryos were isolated from 4 hour collections of *Rho1^E(br)246^/CyO, P{w^+^, Dfd-EYFP}*, *Rho1^E(br)233^/CyO, P{w^+^, Dfd-EYFP}*, or *w^1118^* that were aged to be 12–16 hours AEL, 16–20 AEL, or 20–24 hours AEL. ∼100 embryos from each collection were dechorionated and lysed in 1X SDS sample buffer [32]. The protein samples were boiled, separated on a 12% SDS-PAGE, and transferred to PVDF membrane (Immun-Blot, Bio-Rad, Hercules, CA) for 1 h at 100 V at 4°C. Blots were blocked in 5% nonfat milk in TBS plus 0.1% Tween-20 for 30 min. at room temperature, and then incubated overnight at 4°C in primary antibody. Anti-Rho1 (p1D9 from the Developmental Studies Hybridoma Bank at the University of Iowa, Iowa City, IA) was used at 1:500 and anti-β-tubulin (E7 from the DSHB) was used at 1:3,000. After incubation with horseradish peroxidase–coupled secondary antibodies (Jackson ImmunoReseach Laboratories, West Grove, PA), the immunoreactive proteins were visualized using chemiluminescent detection (Pierce, Rockford, IL).

### Deficiency screen

Second-site noncomplementation (SSNC) tests between autosomal deficiencies (or specific mutations) and *Rho1^E(br)246^* were performed by mating eight to ten *Rho1^E(br)246^/CyO, P{w^+^, ActGFP}* virgin females to eight to ten deficiency– or specific mutation–bearing heterozygous males in vials. After 3 days the adults were transferred to fresh vials, and then to a third vial after two additional days. Newly eclosing F_1_ flies were separated by genotype and examined for malformed legs each day for a total of 10 days per vial. Subsequent secondary screening with *Rho1^E(br)233^* and *Rho1^E3.10^* were performed in the same manner, as were SSNC tests between *Rho1*-interacting deficiencies or specific *Rho1*-interacting mutations and mutations in *RhoGEF2* and *zip*. SSNC tests involving X-linked deficiencies or mutations were performed in a similar manner, but we reversed the sexes of the crossed stocks (i.e. *Rho1^E(br)246^/CyO, P{w^+^, ActGFP}* males crossed to deficiency-bearing hemizygous virgin females).

**Figure 1:**
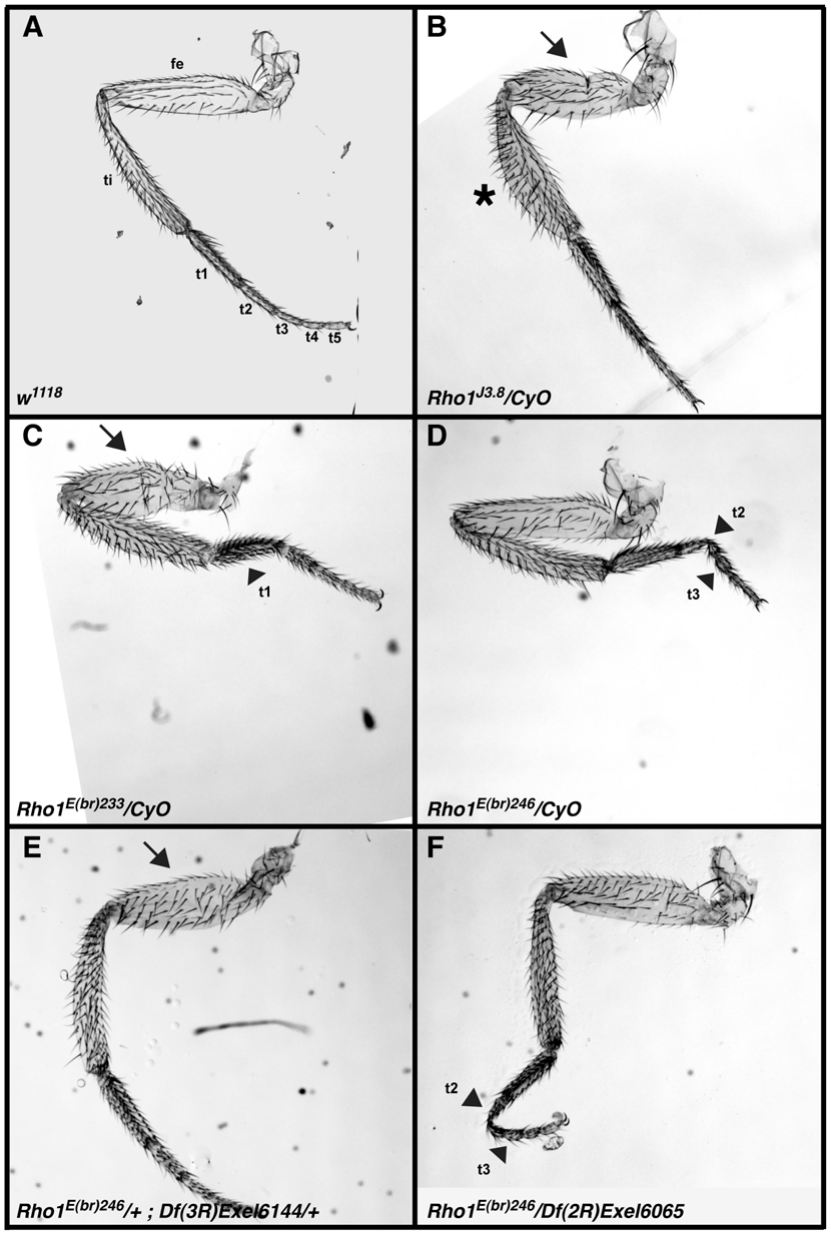
Representative malformed leg phenotypes in animals heterozygous for mutations in *Rho1*, or doubly heterozygous for mutations in *Rho1^E(br)246^* and specific *Rho1*-interacting deficiencies. Brightfield photomicrographs of representative adult legs from the third thoracic segment of *w^1118^* (A), *Rho1^J3.8^/Cyo* (B), *Rho1^E(br)233^/CyO* (C), *Rho1^E(br)246^/CyO* (D), *Rho1^E(br)246^/+;Df(3R)Exel6144/+* (E), and *Rho1^E(br)246^/Df(2R)Exel6065* (F). Femur (fe), tibia (ti), and the five tarsal segments (t1-t5) are labeled in (A). Some animals show short, fat femurs (arrows in B, C and E) or tibias (asterisk in B), whereas others show tarsal segments that are short and fat, or are long and thin and occasionally show severe bends (arrowheads in C, D and F). Note that malformations in all leg segments are seen with each *Rho1* allele, and in the interaction between *Rho1* alleles and specific *Rho1*-interacting deficiencies.

Prior to conducting the screen we considered five alleles of *Rho1* (*Rho1^E(br)233^*, *Rho1^E(br)246^*, *Rho1^k02107b^*, *Rho1^J3.8^* and *Rho1^E3.10^*) and tested them with mutations in two known *Rho1*-interacting genes, *zip* and *RhoGEF2*, at 21°C and 25°C. We considered an animal to be malformed if it displayed even a single malformed leg, and defined a leg as malformed if any femur, tibia or tarsal segment was bent or twisted or was excessively short and fat (examples are shown in Figure 1B–D). As shown in Table 1, only *Rho1^E(br)246^/+* and *Rho1^E3.10^/+* showed a background penetrance of malformed legs less than 2% at either temperature (*Rho1^E(br)246^/+* at 21°C and *Rho1^E3.10^/+* at both temperatures). Both of these *Rho1* alleles are also capable of being strongly enhanced by heterozygous mutations in *RhoGEF2* and *zip*, with *Rho1^E(br)246^* showing a modestly better interaction. Given the fact that *Rho1^E(br)246^* is an amorphic allele, whereas *Rho1^E3.10^* is likely an antimorphic allele [23], we decided to use *Rho1^E(br)246^* for the screen. In addition, although we observed stronger interactions at 25°C than 21°C for all the *Rho1* alleles, we decided to conduct the screen at 21°C since the background level of malformations was lower at this temperature, and thus would likely maximize our ability to identify *Rho1*-interacting loci. Using these conditions, we established our threshold for interaction at 10% malformed legs in animals doubly heterozygous for *Rho1^E(br)246^* and any deficiency or specific mutation.

**Table 1:** SSNC control data with select *Rho1* alleles.

Genotype^a^	Temperature (°C)	%Malformed (n)^b^
Rho1^E(br)246^/+	21	1.6 (689)
	25	3.1 (878)
Rho1^E(br)233^/+	21	3.1 (709)
	25	4.9 (733)
Rho1^E3.10^/+	21	1.0 (295)
	25	0.7 (277)
Rho1^k02107b^/+	21	4.7 (172)
	25	4.1 (195)
Rho1^J3.8^/+	21	2.0 (406)
	25	3.1 (425)
Rho1^E(br)246^ +/+ RhoGEF2^11-3b^	21	37 (252)
	25	86 (43)
Rho1^E(br)233^ +/+ RhoGEF2^11-3b^	21	31 (239)
	25	75 (61)
Rho1^E3.10^ +/+ RhoGEF2^11-3b^	21	20 (143)
	25	20 (30)
Rho1^k02107b^ +/+ RhoGEF2^11-3b^	21	80 (5)
	25	100 (9)
Rho1^J3.8^ +/+ RhoGEF2^11-3b^	21	55 (62)
	25	91 (22)
Rho1^E(br)246^ +/+ zip^E(br)^	21	50 (82)
	25	66 (44)
Rho1^E(br)233^ +/+ zip^E(br)^	21	20 (102)
	25	66 (29)
Rho1^E3.10^ +/+ zip^E(br)^	21	ND
	25	33 (12)
Rho1^k02107b^ +/+ zip^E(br)^	21	ND
	25	ND
Rho1^J3.8^ +/+ zip^E(br)^	21	95 (20)
	25	97 (30)

a Balanced, *Rho* heterozygous mutant virgin females were crossed to either *w^1118^* males, or males bearing *RhoGEF2* or *zip* mutations over a second chromosome balancer at 21°C and 25°C. ^b^% malformed indicates the percentage of animals of the indicated genotype showing a malformed leg phenotype in at least one leg. *n*, total number of flies of the indicated genotype that were scored. ND, not determined.

For the primary screen we used the Exelixis collection of deficiencies maintained by the Bloomington *Drosophila* Stock Center. It has been reported that these deficiencies collectively uncover ∼56% of the *Drosophila* genome (predicted genes; [27]), although complementation tests conducted by the *Drosophila* stock center have shown that at least 10% are not completely deficient for the indicated intervals (http://flystocks.bio.indiana.edu/Browse/df-dp/dfextract.php?num=all&symbol=exeldef), and thus the collection provides closer to 50% coverage. It should be noted that two of the stocks that passed our primary screen, *Df(1)Exel8196* and *Df(1)Exel6253*, were not tested by the stock center, but all of the remaining deficiencies that we describe in the text have been confirmed by the stock center.

### Adult specimen preparations

Adult leg cuticles were prepared by dissecting legs from the third thoracic segment of *w^1118^*, *Rho1^E(br)246^/+*, *Rho1^E(br)233^/+*, *Rho1^J3.8^/+*, *Rho1^E(br)246^/+;Df(3R)Exel6144, or Rho1^E(br)246^/Df(2R)Exel6065* in PBS, clearing them overnight in 10% KOH, and mounting them in Euporal (Bioquip, Gardena, CA) on microscope slides. Images of adult leg cuticles were captured on a Photometrics CoolSNAP *ES* high performance digital CCD camera mounted on a Nikon Eclipse 80*i* microscope. Images of adult legs from live wild type flies and flies overexpressing Tor signaling pathway genes were captured on Photometrics CoolSNAP *cf* color digital CCD camera mounted on a Leica MZFLIII stereomicroscope. All digital images were cropped and adjusted for brightness and contrast in Adobe Photoshop (version CS3, San Jose, CA).

## Results

### Characterization of newly isolated *Rho1* alleles

**Figure 2:**
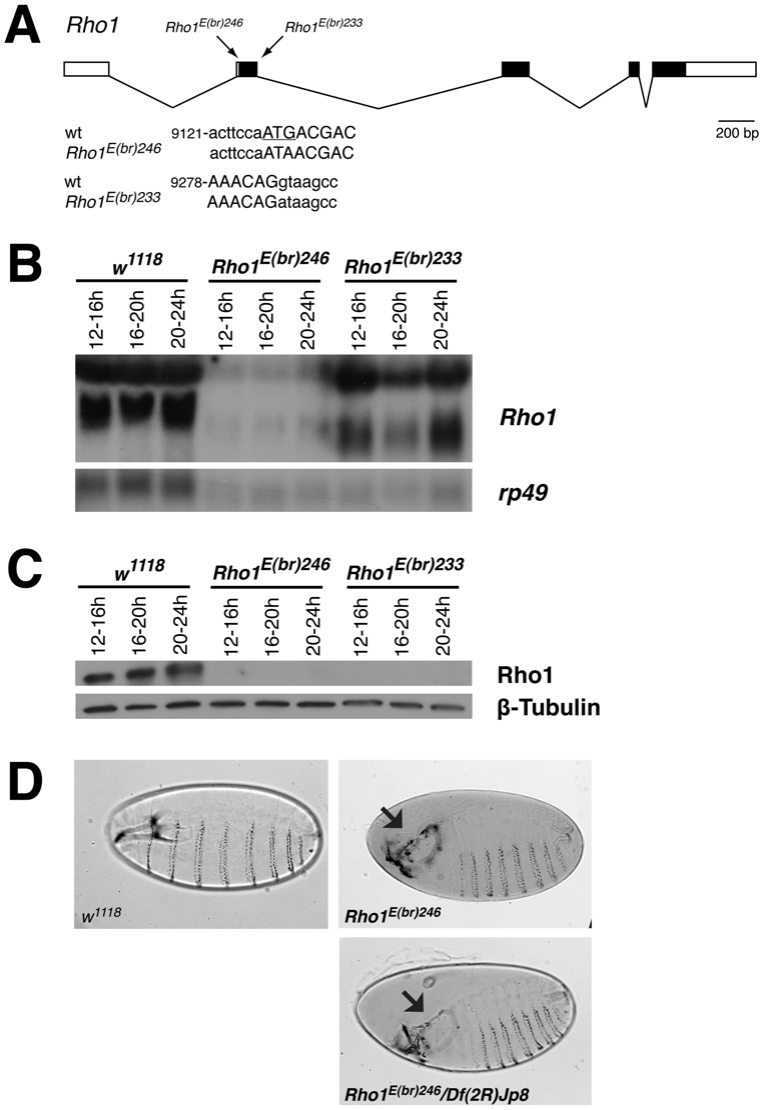
Molecular and genetic characterization of *Rho1^E(br)246^* and *Rho1^E(br)233^*. (A) Schematic diagram of the *Rho1* transcript. Exons are indicated by boxes with coding regions filled in black. The locations of the *Rho1^E(br)246^* and *Rho1^E(br)233^* mutations are indicated above the diagram and the molecular lesions are shown in the sequences below. 5′ untranslated sequence and intronic sequences are written in lowercase letters while coding sequence is written in capital letters. The ATG start codon is underlined. The number refers to the sequence from genomic clone AF177871. (B) Northern blot analysis of total RNA isolated from *w^1118^*, *Rho1^E(br)246^*, and *Rho1^E(br)233^* mutant embryos at 12–16 hrs, 16–20 hrs, and 20–24 hrs after egg laying. Hybridization to *rp49* was used as a control for loading and transfer, and indicates that the *Rho1* samples are underloaded relative to *w^1118^*. Hybridization of the blot with *Rho1* indicates that *Rho1^E(br)246^* produces substantially less *Rho1* transcripts, whereas *Rho1^E(br)233^* produces transcripts at ∼ wild type levels but with altered size. The two prominent bands in the *w^1118^* samples are the 2.1 kb and 1.3 kb transcripts described by [12]. (C) Western blot analysis of total protein lysate isolated from *w^1118^*, *Rho1^E(br)246^*, and *Rho1^E(br)233^* mutant embryos at 12–16 hrs, 16–20 hrs, and 20–24 hrs after egg laying. No Rho protein is observed in *Rho1^E(br)246^* or *Rho1^E(br)233^* mutant embryos. β-Tubulin was used as a control for loading and transfer. (D) Brightfield photomicrographs of cuticle preparations of *w^1118^*, *Rho1^E(br)246^* and *Rho1^E(br)246^/Df(2R)Jp8* mutant embryos. All animals are shown with anterior to the left and the dorsal surface up. All *Rho1^E(br)246^* and *Rho1^E(br)246^/Df(2R)Jp8* mutant animals die as embryos, with nearly completely penetrant defects in head involution (arrows point to dorsal anterior holes in the cuticle).

Molecular characterizations of two new EMS-induced mutations of *Rho1* that we recovered from a modifier screen of *br^1^* [25] demonstrate that they are null alleles. Sequence analysis of genomic DNA from *Rho1^E(br)246^* embryos revealed a G/C to A/T transition in the start codon (nucleotide 9130 from genomic clone AF177871; Figure 2A). Consistent with this observation, we did not detect any Rho1 protein by western blot or by indirect immunofluorescence of fixed embryos in *Rho1^E(br)246^* mutant animals from 12 hr after egg laying (the earliest time point we could unambiguously identify mutant embryos; Figure 2C and data not shown). We also observed reduced *Rho1* transcript levels in mutant embryos (Figure 2B), raising the possibility that this mutation engages a nonsense-mediated RNA decay pathway. Similarly, genomic sequencing of *Rho1^E(br)233^* revealed a G/C to A/T transition at the invariant G in the splice donor site of the first exon (nucleotide 9284 from AF177871; Figure 2A). Northern blot analysis of total RNA isolated from 12–24 hour *Rho1^E(br)233^* mutant embryos revealed nearly wild type levels of expression, but altered *Rho1* transcript sizes, consistent with a defect in splicing (Figure 2B). We have not determined the nature of the altered transcript since there are several potential splice donor sites in the 1.2 kb intron. The protein predicted from this allele would encode the amino terminal 52 amino acids of Rho1 followed by 12 novel (non-Rho1) amino acids before a premature stop codon. Consistent with this result we do not detect any Rho1 protein by western blot or by indirect immunofluorescence of fixed embryos in *Rho1^E(br)233^* mutant animals (Figure 2C and data not shown), although the 1D4 anti-Rho1 monoclonal antibody recognizes an epitope within the carboxyl-terminal 55 amino acids [33], and therefore would not detect the mutant protein. Together, these molecular characterizations suggest that *Rho1^E(br)246^* and *Rho1^E(br)233^* are likely null alleles.

Genetic experiments on *Rho1^E(br)246^* and *Rho1^E(br)233^* support the conclusions of the molecular data and demonstrate that they are amorphic alleles. *Rho1^E(br)246^* mutant embryos show complete embryonic lethality with a nearly completely penetrant defect in head involution (135/136 mutant embryos showed anterior open defects). The phenotype is identical in penetrance and expressivity to *Rho1^E(br)246^/Df(2R)Jp8* (Figure 2D), and is consistent with the zygotic loss of function phenotype reported for the strong loss of function *Rho1* allele reported by Magie et al. [12]. Similarly, *Rho1^E(br)233^* mutant embryos show complete embryonic lethality with nearly completely penentrant head involution defects (data not shown).

### A second-site noncomplementation (SSNC) screen for modifiers of Rho signaling during leg morphogenesis

In order to identify genes that function as part of a Rho signaling pathway required for leg imaginal disc morphogenesis during metamorphosis, we screened the Exelixis deficiency collection [27] for deficiencies that increased the penetrance of malformed legs in animals doubly heterozygous for the deficiency and a loss of function allele of *Rho1* (Examples of malformed legs in animals doubly heterozygous for *Rho1^E(br)246^* and specific deficiencies are shown in Figure 1E and F). The Exelixis collection consists of ∼500 molecularly-defined deficiencies generated by FLP-mediated recombination of FRT-bearing *P*-element stocks. The key advantages to this collection of deficiencies are that they were generated in a near isogenic background, the deletions are small (∼140 kb on average), and the breakpoints are known. Prior to the screen we conducted a series of control experiments to determine which *Rho* allele and what conditions were best for conducting the large-scale screen (details are presented in Materials and Methods). It should be noted that all of the potential *Rho1* alleles show a dominant, partially penetrant malformed leg phentoype that varies between 1 and 5 percent depending upon the allele and temperature of development (Table 1; Figure 1B–D). From these experiments we chose to use *Rho1^E(br)246^* at 21°C for the screen, and established a threshold for interaction at 10% malformed legs in animals doubly heterozygous for *Rho1^E(br)246^* and any deficiency or specific mutation. Of the 461 deficiency stocks tested in the primary screen, 18 reached this threshold (there were two exceptions in which we observed an overall penetrance of 8%, but at least one of the three vials tested showed greater than 10% and we had additional evidence supporting the interval; see below). The entire dataset is presented in Table S1.

Through extensive secondary screening we confirmed 12 regions as containing putative *Rho1*-interacting genes and refined the intervals containing these genes (Table 2; the entire dataset including all SSNC tests with deficiencies and specific mutations for intervals that passed the primary screen is presented in Table S2). To accomplish this we retested the 18 deficiencies that passed the primary screen, along with additional overlapping deficiencies, for SSNC with *Rho1^E(br)246^*, and then tested the primary deficiency with two additional *Rho1* alleles (*Rho1^E(br)233^* and *Rho1^E3,10^*). After these secondary tests, we considered that a region contains a *Rho1*-interacting gene if the primary deficiency interacted with at least two alleles of *Rho1* and at least two overlapping deficiencies interacted with an allele of *Rho1* (one exception to this rule was *Df(1)Exel6253* in which overlapping deficiencies were not available, see below). It should be noted that for the twelve deficiencies that passed these secondary tests there was low variability in the penetrance of malformed legs between the primary screen and the subsequent retest. We first determined the mean penetrance of malformed legs and the standard error of measurement. We then calculated the standard error as a percentage of the mean. Overall there was 18% variance around the mean for all of these deficiencies, with a range from 0% of the mean for *Df(3R)Exel6144* to 34% of the mean for *Df(3R)Exel7328*.

**Table 2:** Summary of *Rho1*-interacting deficiencies and specific mutations.

			% malformed (n)^c^		
Primary screen Df^a^	Secondary screen Df or specific mutation	Cytology^b^	Rho1^E(br)246^	Rho1^E(br)233^	Rho1^E3.10^
**Df(1)Exel8196**		2B1; 2B5	9 (78)	31 (42)	50 (28)
	Df(1)A94	1E3; 2B12	48 (23)		
	br^5^	2B3-5	9 (33)**^d^**	8 (49)**^d^**	
	br^1^	2B3-5	18 (57)**^d^**	21 (38)**^d^**	62 (53)**^d^**
	dor^8^	2B5	11 (61)		
**Df(1)Exel6245**		11E11; 11F4	11 (46)	11 (47)	0 (84)
	Df(1)N12	11D; 11F2	13 (82)		
	Df(1)C246	11D1; 12A1	13 (38)		
**Df(1)Exel6253**		18D13; 18F2	11 (85)	25 (51)	18 (105)
	Cdc42^1^	18E1	52 (40)	47 (34)	47 (38)
	Cdc42^3^	18E1	9 (99)	13 (68)	27 (30)
**Df(2L)Exel6017**		27E4; 27F5	19 (54)	3 (124)	1 (107)
	Df(2L)spd^j2^	27B2; 27F2	13 (122)		
	Df(2L)ED489	27E4; 28B1	12 (78)		
**Df(2L)Exel7055**		34A2; 34A7	15 (65)	10 (52)	11 (105)
	Df(2L)prd1.7	33B3; 34A2	0 (172)		
	Df(2L)ED776,	33E4; 34A3	1 (136)		
	Df(2L)ED777	33E7; 34A3	0 (72)		
	Df(2L)ED773	33E9; 34A3	0 (177)		
	Df(2L)ED778	33E9; 34A7	18 (89)		
	Df(2L)Exel8028	34A1; 34A2	0 (135)		
	Df(2L)ED774	34A3; 34A3	0 (121)		
	Df(2L)BSC30	34A3; 34B9	15 (89)		
	Df(2L)ED784	34A4; 34B6	32 (31)		
	Df(2L)Exel9023	34A6; 34A7	0 (211)		
	Tor^DeltaP^	34A4	5 (354)	5 (195)	5 (129)
	P{lacW}Tor^K17004^	34A4	4 (161)		
**Df(2R)Exel7098**		44D5; 44E3	10 (71)	14 (125)	9 (183)
	Df(2R)ED1742	44B9; 44E3	11 (149)		
	Df(2R)H3D3	44D1; 44F5	8 (77)		
	Df(2R)ED1770	44D8; 45B4	55 (60)	60 (58)	23 (64)
**Df(2R)Exel6065**		53D14; 53F9	17 (163)	34 (127)	50 (70)
	Df(2R)ED2751	53D14; 53F9	38 (128)		
	Df(2R)ED1	53E4; 53F9	31 (80)		
	PBac{RB}RhoGEF2^e03784^	53E4-F1	57 (28)		
	P{EPgy2}RhoGEF2^EY08391^	53E4-F1	17 (92)		
	RhoGEF2^11-3b^	53E4-F1	37 (252)		
**Df(2R)Exel6098**		63F2; 63F7	8 (120)	8 (76)	10 (72)
	Df(3L)ED208	63C1; 63F5	23 (59)	35 (62)	56 (19)
	Df(3L)ED4341	63F6; 64B9	10 (63)		
	Sc2^1^	63F5	14 (133)	33 (131)	11 (80)
	P{PZ}Sc2^05634^	63F5	3 (139)		
	Sc2^A4^	63F5	4 (92)		
	Sc2^F9^	63F5	0 (95)		
	P{UASp-YFP.Rab8.Q67L}Sc2^10^	63F5	0 (126)		
**Df(3R)Exel6144**		83A6; 83B6	10 (249)	25 (87)	5 (111)
	Df(3R)ED5177	83B4; 83B6	0 (61)		
	P{Mae-UAS.6.11}Rheb^AV4^	83B2	8 (130)	14 (77)	4 (113)
	P{SUPor-P}Rheb^KG02006^	83B2	3 (149)		
	P{EPgy2}Rheb^EY08085^	83B2	2 (133)		
	P{Mae-UAS.6.11}Rheb^LA01053^	83B2	4 (162)		
**Df(3R)Exel7328**		89B1; 89B9	8 (227)	37 (84)	55 (116)
	Df(3R)Exel7327	89A8; 89B3	3 (95)		
	Df(3R)bxd100	89B6; 89E2	18 (89)		
	Sbd^E(br)536^	89B4-6			14 (72)^d^
**Df(3R)Exel6178**		90E7; 91A5	18 (212)	42 (85)	18 (152)
	Df(3R)P14	90C2; 91B2	9 (68)	6 (82)	14 (59)
	Df(3R)Cha7	90F1; 91F5	11 (76)		
	Df(3R)ED5815	90F4; 91B8	8 (75)		
**Df(3R)Exel6179**		91A5; 91B5	12 (272)	21 (112)	11 (159)
	Df(3R)Cha1a	91A2; 92A1	28 (46)		
	Df(3R)ED2	91A5; 91F1	15 (127)	28 (25)	
	Df(3R)BX5	91B1; 91D2	4 (134)		
	Df(3R)07280	91B2; 91C1	13 (125)		

a Exelixis deficiencies identified in the primary screen that interact with more than one *Rho1* allele and have been confirmed by the identification of overlapping *Rho1*-interacting deficiencies. ^b^Cytology is based upon flybase annotations as of January 2009 (reflects release 5 of the *Drosophila* genome). ^c^% malformed indicates the percentage of animals heterozygous for the indicated *Rho1* allele and heterozygous for the indicated deficiency or specific mutation showing the malformed leg phenotype in at least one leg. *n*, total number of flies of the indicated genotype that were scored. ^d^Data from [25].

Three of these interacting regions contain the previously identified *Rho1*-interacting genes *broad*, *RhoGEF2*, and *stubbloid*, and we have strong genetic evidence that another of the interacting intervals is due to an interaction between *Rho1* and *Cdc42*. Further, our results indicate that *Rheb* and *Sc2* interact genetically with *Rho1* during imaginal disc morphogenesis, and that the *Target of rapamycin* (*Tor*) may also interact with *Rho1* during this process. The details of these interactions are presented below.

#### broad (br)

*Df(1)Exel8196* is predicted to uncover 18 genes from cytological region 2B1 to 2B5, including *br*. Although the deficiency stock was sick and we consistently obtained only small numbers of *Df/+;Rho1^E(br)246^/+* animals, we did observe malformed legs at a frequency that placed this interval above the threshold in the majority of the vials tested. Consistent with this observation, *Df(1)Exel8196* also showed SSNC with *Rho1^E(br)233^* and *Rho1^E3.10^* (Table 2). Furthermore, a larger overlapping deficiency, *Df(1)A94*, also showed SSNC with *Rho1^E(br)246^*. Since we previously identified *Rho1^E(br)246^* and *Rho1^E(br)233^* as dominant modifiers of the malformed leg phenotype associated with *br^1^*, and demonstrated that both *Rho* alleles show SSNC with the amorphic allele *br^5^* [25], we are confident that the genetic interaction observed with this interval is due to an interaction between *Rho1* and *br*. It should be noted that *dor^8^* also showed SSNC with *Rho1^E(br)246^* (Table 2), raising the possibility that *deep orange* may also be a *Rho1*-interacting gene, although thus far we have not followed up on this observation.

#### RhoGEF2

*Df(2R)Exel6065* is a large molecularly defined deficiency that uncovers 47 predicted genes in the cytological interval 53D14 to 53F9. This deficiency displayed SSNC with all three alleles of *Rho1* tested (Table 2). In addition, *Df(2R)ED2751*, a molecularly defined deficiency whose breakpoints are each within 200 bp of those for *Df(2R)Exel6065*, also shows SSNC with *Rho1^E(br)246^*. In order to refine this interval we tested *Df(2R)ED1* and observed a robust genetic interaction with *Rho1^E(br)246^*. This molecularly defined deficiency has a left breakpoint within the first intron of *RhoGEF2* and a right breakpoint identical to *Df(2R)Exel6065*. In total this deficiency is predicted to uncover 14 genes. There were loss of function alleles for four of these genes and we tested all of them for interaction with *Rho1^E(br)246^*. We observed SSNC with multiple *RhoGEF2* alleles, but with none of the other mutations (Table 2 and Table S2). The identification of *RhoGEF2* as a modifier for *Rho1* during imaginal disc morphogenesis supports earlier observations from Halsell and Kiehart [23] and Bayer et al. [26].

#### Stubbloid (sbd)

The final previously identified *Rho1*-interacting gene we identified through the primary screen was *sbd*. *Df(3R)Exel7328* showed 8% malformed legs in the primary screen, but several of the individual vials showed greater than 10% malformations. We therefore tested this deficiency with *Rho1^E(br)233^* and *Rho1^E3.10^* and observed much stronger interactions (Table 2). This deficiency is predicted to uncover 29 genes between 89B1 and 89B9. From the primary screen, we determined that an overlapping deficiency, *Df(3R)Exel7327*, does not show SSNC with *Rho1^E(br)246^*. *Df(3R)Exel7327* has a left breakpoint in 89A8 and a right breakpoint in 89B3, thereby limiting the interacting interval to 19 genes including *sbd*. An overlapping deficiency, *Df(3R)bxd100* (predicted interval: 89B6-89E2), also shows SSNC with *Rho1^E(br)246^*, strongly supporting this interval. Although we did not test any of the other genes in this interval, we had previously shown a genetic interaction between *sbd* and *Rho1* [25], as had Bayer et al. [26].

#### Cdc42

We observed a SSNC between *Df(1)Exel6253* (predicted interval: 18D13-18F2) and *Rho1^E(br)246^*, *Rho1^E(br)233^* and *Rho1^E3.10^*. Although we were not able to confirm this finding with an overlapping deficiency that also showed SSNC with *Rho1^E(br)246^*, we were able to test loss of function alleles for 6 of the 32 genes predicted for this interval (all that had loss of function alleles available from the Bloomington *Drosophila* stock center), and found SSNC between two alleles of *Cdc42* and all three tested alleles of *Rho1*. The interaction was stronger with *Cdc42^1^* than with *Cdc42^3^*. In fact, *Cdc42^1^* showed a greater interaction with *Rho1^E(br)246^* than did *Df(1)Exel6253*, and produced very few *Cdc42^1^/+; Rho1^E(br)246^/+* animals, although the other classes of offspring were well represented. This observation is consistent with a report that *Cdc42^1^* is an antimorphic allele [14].

#### Rheb

During the primary screen we observed SSNC between *Df(3R)Exel6144* (predicted interval 83A6-83B6) and both *Rho1^E(br)246^* and *Rho1^E(br)233^*. The *Rho1*-interacting region was further refined by the lack of interaction between *Rho1^E(br)246^* and the molecularly defined deficiency *Df(3R)ED5177* (predicted interval 83B4-83B6), thereby limiting the region to 17 predicted protein-coding genes and 16 small nucleolar RNA genes in 83A6-83B4. We were able to test lethal alleles for eight of these genes, seven of which showed no SSNC with *Rho1^E(br)246^* (Table S2). The final mutation, *P{Mae-UAS.6.11}Rheb^AV4^*, showed SSNC with both *Rho1^E(br)246^* and *Rho1^E(br)233^* (Table 2). This allele of *Rheb* is a P-element insertion that has an UAS element to allow for overexpression of *Rheb*, but the insertion also acts as a recessive lethal allele of *Rheb*, resulting in slow growth and eventual death as delayed first instar larvae [34]. Two additional P element alleles of *Rheb*, *P{Mae-UAS.6.11}Rheb^LA01053^* and *P{EPgy2}Rheb^EY08085^*, failed to show SSNC with *Rho1^E(br)246^* (Table 2).

#### Sc2

We observed a weak SSNC between *Df(3L)Exel6098* (predicted interval 63F2-63F7) and *Rho1^E(br)246^* during the primary screen. We observed a similar interaction between this deficiency and *Rho1^E(br)233^* and *Rho1^E3.10^*. During the secondary screen we observed a very strong SSNC between *Rho1^E(br)246^* and *Df(3L)ED208*, a molecularly defined deficiency with breakpoints in 63C1 and 63F5. We also observed SSNC between *Rho1^E(br)246^* and *Df(3L)ED4341*, another molecularly defined deficiency (predicted interval: 63F6-64B9) that overlaps with *Df(3L)Exel6098*, but not with *Df(3L)ED208*, suggesting that there are likely two genes responsible for these interactions (Table 2). One of these genes is predicted to map to interval 63F6-7 that contains only four genes, *Sc2*, *ida*, *mge*, and *Eip63F-1*. We therefore tested loss of function alleles for *Sc2*, *ida* and *mge* (all of the *Eip63F*-1 alleles are viable) and found SSNC between *Sc2^1^* and *Rho1^E(br)246^*, *Rho1^E(br)233^* and *Rho1^E3.10^* (Table 2 and Table S2). *Sc2^1^* is an EMS generated hypomorphic allele resulting in pupal lethality [35]. The other three alleles of *Sc2*, however, showed 3-4% malformed legs when heterozygous with *Rho1^E(br)246^/+*, and thus were deemed to not interact. To identify the other potential *Rho1*-interacting gene we tested mutations in 13 genes that mapped to the interval 63C1 to 63F5 for SSNC with *Rho1^E(br)246^* (representing all of the genes that had loss of function mutations available from the stock center). None of these mutations, however, showed an interaction with *Rho1^E(br)246^* (Table S2).

#### Tor

We identified a SSNC between *Rho1^E(br)246^* and *Df(2L)Exel7055* (predicted interval 34A2-34A7). *Df(2L)Exel7055* also showed SSNC with *Rho1^E(br)233^* and *Rho1^E3.10^*. To refine this interval we tested ten additional deficiencies with three of them interacting and seven failing to interact (Table 2). Using the molecularly defined deficiencies, we were able to limit the *Rho1*-interacting region to 13 potential genes between *Target of rapamycin (Tor)* and *Sir2* in 34A4-34A7. We tested loss of function alleles for five of these genes (all that were available), but did not observe a genetic interaction with any of them (Table S2). We did, however, observe an interaction between *Tor^ΔP^* and *Rho1^E(br)246^* that, although below our threshold, consistently gave 5% malformed legs with all three tested *Rho1* alleles. *Tor^ΔP^* is a deletion resulting from an imprecise excision of a P-element that removes the start codon and the amino terminal 902 codons of Tor and thus is likely an amorphic allele [36]. We tested a weaker loss of function allele of *Tor*, *P{lacW}Tor^k17004^*, but observed less than 5% malformed legs with *Rho1^E(br)246^* (Table 2).

#### Other *Rho1*-interacting loci

There are five additional intervals that are predicted to contain *Rho1*-interacting genes based upon the criteria outlined above: 11E11-11F2, 27E4-27F2, 44D8-44E3, 90F4-91A5, and 91A5-91B1. We have narrowed these intervals as far as possible using all available molecularly defined deficiencies, and in each case have tested putative loss of function mutations for all the genes for which stocks are available, but have not found any additional potential *Rho1*-interacting genes (Table S2). Although we have carefully examined the lists of genes for these remaining intervals, there are no obvious candidates for *Rho1*-interacting genes, and thus the cloning and characterization of these genes should provide novel insights into Rho1 signaling during leg development.

### Genetic interactions between *Rho1*-interacting deficiencies and components of the Rho signaling pathway

In order to further characterize the *Rho1*-interacting deficiencies, we crossed all of them (and several of the putative *Rho1*-interacting specific mutations) to *zip^E(br)^* and *RhoGEF2^11-3b^*, and found that nine of the twelve deficiencies showed SSNC with at least one of these mutations. This experiment was predicated on previous studies reporting strong genetic interactions between *Rho1* and several components in the Rho signaling pathway, including *RhoGEF2* and *zip*, during leg imaginal disc development [23]–[26]. We first determined the background level of malformed legs in *RhoGEF2^11-3b^/+* and *zip^E(br)^/+* adults. *RhoGEF2^11-3b^/+* adults showed malformed legs with a frequency of 1% (*n* = 398) regardless of the direction of the cross (for example, *w^1118^* males crossed to *RhoGEF2^11-3b^/CyO* females), whereas the frequency of malformations in *zip^E(br)^/+* varied according to the sex of the parents in the cross, with a higher frequency of malformed progeny resulting from a cross in which the mother provided the *zip^E(br)^* allele (2%, *n* = 126 with *zip^E(br)^/SM5* males and 6%, *n* = 116 with *zip^E(br)^/SM5* females). Since all of the autosomal deficiency crosses were set up with *zip^E(br)^/SM5* mothers, we considered an interaction to be significant when the progeny showed 20% or greater malformations, whereas we retained the 10% threshold for all other crosses. As shown in Table 3, seven of the twelve *Rho1*-interacting deficiencies showed SSNC with both *zip^E(br)^* and *RhoGEF2^11-3b^* (or failed to complement *RhoGEF2* in the case of *Df(2R)Exel6065*), strongly implicating genes uncovered by these deficiencies in Rho signaling. It should be noted that *Df(2R)Exel7328*, which removes *sbd* and showed the strongest SSNC with *RhoGEF2^11-3b^*, was not tested for interactions with *zip^E(br)^*, since genetic interactions between *sbd* and *zip* had been well documented [25], [26]. Two of the five remaining deficiencies showed SSNC with *zip^E(br)^*, but not with *RhoGEF2^11-3b^*.

**Table 3:** SSNC tests between *Rho1*-interacting deficiencies and *zip* and *RhoGEF2.*

			% malformed (n)^b^	
Primary screen Df	Specific mutation	Cytology^a^	zip^E(br)^	RhoGEF2^11-3b^
**Df(1)Exel8196**		2B1; 2B5	59 (39)	26 (14)
	br^1^	2B5	24 (88)	ND
**Df(1)Exel6245**		11E11; 11F4	13 (45)	3 (61)
**Df(1)Exel6253**		18D13; 18F2	13 (45)	24 (38)
	Cdc42^1^	18 E1	64 (36)	34 (47)
	Cdc42^3^	18 E1	8 (114)	4 (112)
**Df(2L)Exel6017**		27E4; 27F5	8 (107)	1 (150)
**Df(2L)Exel7055**		34A2; 34A7	33 (138)	10 (173)
	Tor^DeltaP^	34A4	9 (91)	3 (119)
**Df(2R)Exel7098**		44D5; 44E3	41 (86)	12 (135)
**Df(2R)Exel6065**		53D14; 53F9	77 (99)	Failed to complement
**Df(3L)Exel6098**		63F2; 63F7	11 (88)	1 (127)
	Sc2^1^	63F5-6	17 (46)	1 (173)
**Df(3R)Exel6144**		83A6; 83B6	52 (61)	5 (148)
	Rheb^AV4^	83B2	19 (69)	9 (126)
**Df(3R)Exel7328**		89B1; 89B9	ND	38 (162)
	sbd^E(br)536^	89B4-6	33 (73)**^c^**	2 (119)**^c^**
**Df(3R)Exel6178**		90E7; 91A5	51 (45)	14 (115)
**Df(3R)Exel7179**		91A5; 91B5	11 (111)	5 (174)

a Cytology is based upon flybase annotations as of January 2009 (reflects release 5 of the *Drosophila* genome). ^b^% malformed indicates the percentage of animals heterozygous for the indicated Exelixis deficiency or specific mutation and heterozygous for *zip^E(br)^* or *RhoGEF2^11-3b^* showing the malformed leg phenotype in at least one leg. *n*, total number of flies of the indicated genotype that were scored. Background penetrance of malformed legs: *RhoGEF2^11-3b^/+* 1% (*n* = 398), *zip^E(br)^/+* 2% (*n* = 126) with *zip^E(br)^/SM5* fathers and 6% (*n* = 116) with *zip^E(br)^/SM5* mothers. ^c^Data from [25].

We next tested specific *Rho1*-interacting mutations and found that *Cdc42^1^* strongly interacted with both *zip^E(br)^* and *RhoGEF2^11-3b^*, whereas *Cdc42^3^* did not interact with these mutations. We also found some indication of an interaction between *Sc2^1^* and *zip^E(br)^*, although it was below the threshold we established. Similarly, we observed a trend suggesting an interaction between *Rheb^AV4^* and both *zip* and *RhoGEF2* alleles, although in both cases the results were just below the threshold. These results were encouraging, however, and more strongly suggested an interaction between *Rheb* and the Rho signaling pathway.

**Figure 3:**
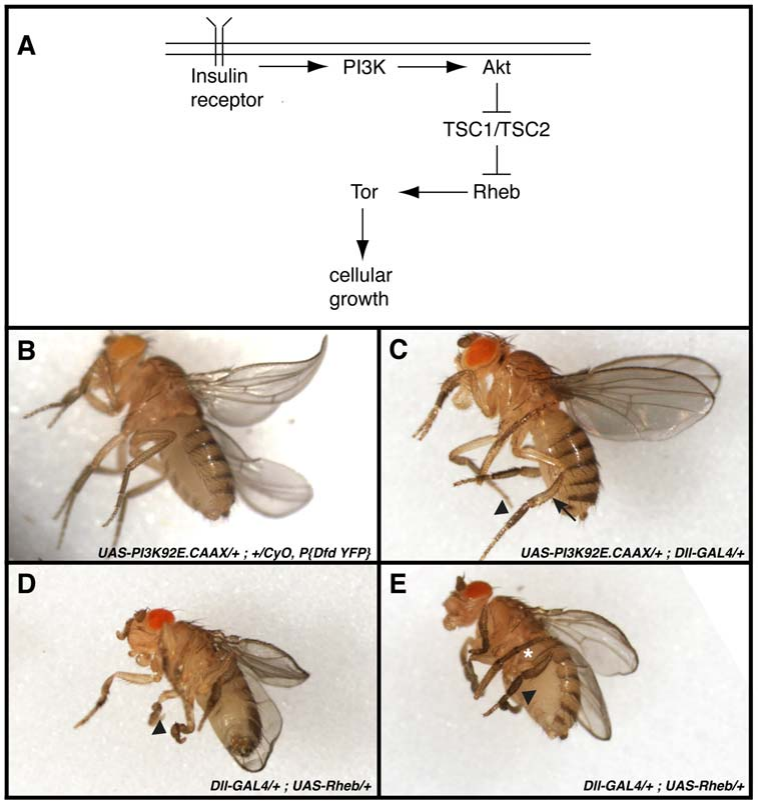
Overexpression of *Rheb* in distal leg segments results in a strongly penetrant malformed leg phenotype. (A) Model of the PI3K/Tor signaling pathway. Details of the pathway are found in the text. (B) Control animals, in this case heterozygous for *UAS-PI3K92E.CAAX*, but without a GAL4 driver, show normal leg morphology in live adults. Similarly, *Dll-GAL4/+* and *UAS-Rheb/+* adults have no leg malformations (not shown). (C) *UAS-PI3K92E.CAAX/+; Dll-GAL4/+* animals express activated PI3K in distal leg segments (distal to the arrow) that results in a growth advantage giving the adults a “Popeye” leg phenotype. The most common morphogenesis defect is a slightly short, fat first tarsal segment (arrowhead). (D and E) *Dll-GAL4/+; UAS-Rheb/+* adults show a highly penetrant malformed leg phenotype independent of the “Popeye” growth phenotype. Mild phenotypes include moderate to extreme short fat distal tibia and tarsal segments (arrowhead in E), whereas more extreme phenotypes include severely misshapen and twisted segments (arrowhead in D). The edges of the wings are often curved while the overall size of the wing is reduced. These defects are often accompanied by severe bends in the femur (white asterisk in E), even though the transgene is not expressed in this segment.

To further address the role of *Rheb* in leg morphogenesis, we overexpressed *Rheb* in distal leg segments using a UAS-GAL4 approach [37] and observed severely malformed legs. *P{GawB}Dll^md23^* flies express the yeast transcription factor GAL4 in the distal half of the tibia and in all the tarsal segments throughout imaginal disc development, as well as in the wing margin, the antennae, and mouth segments (see [38] for the expression pattern of *dll-GAL4* in prepupae and pupae). We crossed two different *UAS-Rheb* transgenic lines to the *P{GawB}Dll^md23^* stock, and in both cases observed offspring with severely malformed legs and wings (100% malformed, *n* = 45 with *P{UAS-Rheb.Pa}2* and 96% malformed, *n* = 170 with *P{UAS-Rheb.Pa}3*; Figure 3D, E). The malformations were characterized by fat tarsal segments that were often nonuniform in diameter and in many cases were kinked or curved. In addition, there was frequently a large kink in the femur, even though the transgene was not expressed in this tissue. The wings tended to be smaller, more delicate and slightly curved. Finally, we noticed a slight bend in the middle tibia corresponding to the boundary of the *Dll* expression domain with the segments distal to this point being larger in general to those proximal to the boundary.

*Rheb* encodes a small GTPase known to activate Tor, which in turn regulates cellular growth via increased protein synthesis and inhibition of autophagy (reviewed in [39]). These proteins act in the highly conserved PI3K/Tor signaling pathway (Figure 3A). PI3 kinase (PI3K) is recruited to the plasma membrane in response to activation of the insulin receptor. At the membrane PI3K catalyzes the conversion of phophatidylinositol 4, 5-bisphosphate into phosphatidylinositol 3, 4, 5-triphosphate (PIP3). Increased levels of PIP3 at the membrane lead to the recruitment of the serine/threonine kinase Akt to the membrane. Akt then negatively regulates the GTPase activating proteins TSC1 and TSC2, which negatively regulate Rheb. Thus recruiting PI3K to the membrane ultimately results in the activation of Tor via Rheb. Since overexpressing *Rheb* may be sufficient to activate Tor and thereby increase growth, we wondered whether these phenotypes were simply due to an increase in cell size for the cells in the *Dll* domain. To address this we expressed *P{UAS-PI3K92E.CAAX}* with *P{GawB}Dll^md23^*. *PI3K92E.CAAX* encodes the *PI3K92E* coding sequence with a farnesylation signal to target the recombinant protein to the membrane and thereby produces a constitutively active kinase. Flies expressing *PI3K92E.CAAX* in the *Dll* domain all showed the enlarged distal tibia and tarsal segments (a phenotype we referred to as “Popeye” legs), but very rarely showed any other malformations. The most extreme malformations we typically observed were the shorter fatter tarsal segments depicted in Figure 3C. Thus the severe malformed leg phenotypes associated with overexpression of *Rheb* are novel and independent of those produced by activating the Tor signaling pathway.

### Assessing potential RhoGEFs involved in leg morphogenesis

*RhoGEF2* is the only potential Rho-specific GEF that definitively showed dose-sensitive interactions with *Rho1* during leg morphogenesis, although two additional genomic intervals containing potential RhoGEF genes also interacted with *Rho1*. Flybase [40] lists 26 genes as having potential RhoGEF activity based upon electronic annotation. Of these, 22 have Dbl homology (DH) domains, and 15 have both DH and PH domains. Since most well-described Rho-specific GEFs possess both a DH and a PH domain [41], we focused our attention on these 15 genes. One of these genes is identified only as a cDNA (GH16492), and thus is not annotated to a particular chromosomal location. We also included *pebble* (*pbl*), a known RhoGEF involved in cytokenesis that has a DH domain and a BRCA1 carboxyl-terminal domain [42]. Of these 15 genes, 7 of them are predicted to be deleted in at least one of the Exelixis deficiencies used in the primary screen. Six of these deficiencies showed no SSNC with *Rho1^E(br)246^*, whereas *Df(2R)Exel6065* showed strong SSNC with *Rho1* and is deficient for *RhoGEF2* (Table 4). To test the remaining 8 potential *RhoGEF* genes, we obtained deficiencies predicted to uncover these genes from the Bloomington *Drosophila* stock center. Two of the deficiencies were molecularly defined, whereas the remaining five were relatively large, randomly generated deficiencies (4 X-ray induced and one generated by imprecise P element excision) with the potential RhoGEF genes well within the predicted endpoints of the deficiencies (*Df(3L)pbl-X1* uncovers both *pbl* and *CG33275*). Three of these seven deficiencies produced at least 10% malformed legs when doubly heterozygous with *Rho1^E(br)246^* (Table 4). *Df(3R)ED5092* showed a SSNC of 12% with *Rho1^E(br)246^* and uncovered the potential RhoGEF *Cdep*. *Cdep* encodes a protein with a FERM domain in addition to DH and PH domains. We failed to detect an interaction with a piggybac allele of *Cdep* (Table 4), although this allele is likely not amorphic.We also observed a very strong SSNC between *Rho1^E(br)246^* and both *Df(2R)nap11* and *Df(2R)ED3636* (Table 4), raising the possibility that CG30440 and/or CG30115 may act as GEFs for Rho1 during leg morphogenesis. Unfortunately there are no specific mutations for either gene, and we have therefore not been able to test these genes directly. We have also thus far not been able to refine these deficiency intervals, although we have tested several specific mutations within the deficiencies and have not identified candidate *Rho1*-interacting genes (data not shown). Interestingly, *Df(2R)nap11* is predicted to uncover genes through 42F including the ecdysone receptor gene *EcR*. Since we observed a strong genetic interaction between the ecdysone-inducible transcription factor *br* and *Rho1*, we suspected that the interaction between *Df(2R)nap11* and *Rho1^E(br)246^* might be due to *EcR*. In contrast to this notion, however, we found no SSNC between *Rho1^E(br)246^* and the amorphic allele *EcR^m554fs^* (data not shown). Finally, *Df(2L)64j* showed an overall interaction with *Rho1^E(br)246^* of 8% and uncovers *Son of Sevenless* (*Sos*), a Ras GEF that also contains DH and PH domains. We therefore tested an amorphic allele of *Sos*, *Sos^34Ea-6^* and found no interaction with *Rho1^E(br)246^* (Table 4).

**Table 4:** SSNC tests to assess potential *RhoGEF* genes.

Potential RhoGEF	Cytology^a^	Protein domains^b^	Df or mutation tested	Cytology^a^	% malf. (*n*)^c^
***CG8557***	16A5-B1	DH, PH	*Df(1)BK10*	15F2; 16C10	0 (91)
***vav***	18B6-7	DH, PH, SH2, SH3, C1, CH	*Df(1)Exel9068*	18B4; 18B6	0 (64)
***Sos***	34D1	DH, PH, RasGEF	*Df(2L)64j*	34D1; 35C1	8 (255)
			*Sos^34Ea-6^*	34D1	2 (133)
***CG10188***	37E4-5	DH, PH	*Df(2L)Exel8041*	37D7; 37F2	0 (106)
***rtGEF***	38C5	DH, PH, SH3	*Df(2L)Exel6046*	38C2; 38C7	5 (21)
***CG30440***	41F2	DH, PH	*Df(2R)nap11*	41E3; 42A10	54 (126)
***RhoGEF2***	53E4-F1	DH, PH, PDZ, C1	*Df(2R)Exel6065*	53D14; 53F9	17 (163)
***CG30115***	55D3-4	DH, PH	*Df(2R)ED3636*	55B8; 55E3	67 (27)
***RhoGEF3***	61B3-C1	DH, PH, SH3	*Df(3L)Exel6084*	61B2; 61C1	6 (242)
***trio***	61E1-2	DH, PH, spectrin	*Df(3L)Exel6086*	61C9; 61E1	0 (208)
***sif***	64E1-5	DH, PH, PDZ	*Df(3L)Exel6106*	64D6; 64E2	4 (116)
***RhoGEF4***	65F4	DH, PH	*Df(3L)BSC33*	65E10;65F6	4 (138)
***pbl***	66A18-19	DH, BRCT	*Df(3L)pbl-X1*	65F3; 66B10	5 (147)
***CG33275***	66A6-8	DH, PH	*Df(3L)pbl-X1*	65F3; 66B10	5 (147)
***Cdep***	82E2-3	DH, PH, FERM	*Df(3R)ED5092*	82A3; 82E8	12 (104)
			*Pbac{5HPw+}Cdep^B122^*	82E2-3	4 (110)

a Cytology is based upon flybase annotations as of January 2009 (reflects release 5 of the *Drosophila* genome). ^b^DH, Dbl homology domain; PH, pleckstrin homology domain; SH2, Src homology 2 domain; SH3, Src homology 3 domain; C1, Protein kinase C conserved region 1 domain; CH, Calponin homology domain; RasGEF, Ras guanine nucleotide exchange factor domain; PDZ, domain found in PSD-95, Dlg and ZO 1/2; BRCT, BRCA1 C-terminal domain; FERM, Protein 4.1, Ezrin, Radixin, Moesin homology domain. ^c^% malformed indicates the percentage of animals heterozygous for the indicated *Rho1* allele and heterozygous for the indicated deficiency or specific mutation showing the malformed leg phenotype in at least one leg. *n*, total number of flies of the indicated genotype that were scored.

## Discussion

### A genetic screen for modifiers of Rho signaling during leg morphogenesis

Using a deficiency-based genetic modifier screen that enabled us to test ∼50% of the genome, we identified 12 chromosomal regions that contain *Rho1*-interacting genes necessary for leg imaginal disc morphogenesis during metamorphosis in *Drosophila*. Through extensive secondary screening we were able to identify the likely *Rho1*-interacting gene for six of these intervals. Three of these genes, *RhoGEF2*, *broad* and *stubbloid*, had previously been shown to interact with *Rho1* during leg development, validating the screen. We additionally identified *Cdc42*, *Rheb* and *Sc2* as new *Rho1*-interacting genes necessary for leg imaginal disc morphogenesis, and observed a possible interaction between *Rho1* and the *Target of rapamycin* (*Tor*) in this process.

Two aspects of the screen greatly contributed to its success. First, we identified an allele of *Rho1*, *Rho1^E(br)246^*, that has a very low background of leg malformations when heterozygous and can be strongly enhanced by second-site mutations in interacting genes. We determined that this is an amorphic allele of *Rho1*, which makes this a truly dose-sensitive interaction screen. The second key to the success of this screen was the use of the Exelixis deficiency collection. The near isogenic background of the stocks in this collection produced essentially no leg malformations, which resulted in a relatively high signal to noise ratio. When we remove the 18 intervals that were deemed to have passed the primary screen, the average percentage of malformed legs in the SSNC class for the remaining lines was 1.8% (as compared to the 1.7% malformations we observed in the cross between *Rho1^E(br)246^* and *w^1118^*; Table 1). Thus, in conjunction with *Rho1^E(br)246^*, the identification of an interacting deficiency with even a modest 6-8 fold increase over background was significant as demonstrated by the fact that 67% of the intervals that were identified in the primary screen were confirmed through the secondary screen. We considered the screen to be successful based upon recovering all possible previously identified *Rho1*-interacting genes involved in leg morphogenesis (*RhoGEF2*; [23], *br*; [25], and *sbd*; [26]). We would have also expected to identify deficiencies uncovering *zip* (in 60E12 on 2R; [24]), and *Rho kinase* (in 14F2 on X; [26]), but the Exelixis collection did not contain deficiencies for these intervals. As a means to further characterize potential *Rho1*-interacting genes we tested each interacting deficiency, as well as specific mutations in candidate genes, for SSNC with alleles of *zip* and *RhoGEF2* (Table 3). Nine of the twelve *Rho1*-interacting deficiencies showed SSNC with at least one of these mutations, as did specific mutations in *br*, *sbd*, and *Cdc42*, with *Rheb* and *Sc2* showing an interaction that was just below our stated threshold (*RhoGEF2* had already been shown to interact with *zip* and was not tested). Taken together, these results indicate that the screen was very successful in identifying genes that play important roles in Rho1 signaling during imaginal disc morphogenesis, and that the cloning and characterization of the remaining *Rho1*-interacting genes will likely provide important novel insights into this critical signaling pathway. We will next describe the newly identified *Rho1*-interacting genes and comment on their role in leg morphogenesis.

#### Cdc42

*Cdc42^1^* showed very strong interactions with all three of the *Rho1* alleles that we tested, as well as with *RhoGEF2^11-3b^* and *zip^E(br)^*. Although *Cdc42^1^* is reported to be an antimorphic allele [14], it does not have a dominant effect on leg morphogenesis (data not shown). In addition, the weaker hypomorphic allele *Cdc42^3^* also showed SSNC with all three alleles of *Rho1*. Together these results support the identification of *Cdc42* as the *Rho1*-interacting gene responsible for the interacting interval identified in 18D-F on the X chromosome.

Cdc42 and Rho1 are closely related small GTPases that likely regulate unique, but complementary processes required for leg morphogenesis. In general, Cdc42 regulates the cytoskeleton by controlling actin polymerization either through the formin mDia2 or by regulating actin branching through WASP and the ARP2/3 complex [43]. Rho can also regulate actin polymerization directly through mDia, but is also likely affecting leg morphogenesis through its regulation of myosin activity, a pathway dependent on Rho kinase. It is therefore possible that during leg morphogenesis Cdc42 regulates the actin cytoskeleton while Rho regulates myosin to affect cell shape changes and cell rearrangements. Another intriguing possibility is that Cdc42 and Rho1 are each regulating distinct aspects of adherens junction plasticity during the cell rearrangements that are occurring during the early stages of leg morphogenesis. Cell rearrangements necessitate the redistribution of junctional material as the border between two adjacent cells either expands or shrinks. It was recently shown that Cdc42 is required for the endocytosis of DE-cadherin and other adherens junction proteins in the early pupal notum, and that it carries out this function in conjunction with Par6 and atypical Protein Kinase C, through its downstream effectors WASP and ARP2/3 [44], [45]. Although the pupal notum is a fairly homeostatic tissue at this time, this function of Cdc42 is likely also critical during the more dynamic stages of tissue morphogenesis occurring 12 hours prior. Rho1 has also been shown to regulate the adherens junction. Specifically, Rho1 localizes to the adherens junction through its interaction with α-catenin and p120 catenin [33], and has been shown to regulate the distribution of DE-cadherin at adherens junctions during embryonic morphogenesis [46], likely through a mechanism dependent upon its downstream effector diaphanous [47].

#### Rheb

During the primary screen we identified a potential *Rho1*-interacting interval in 83A-B that we were not able to verify with an overlapping deficiency, although we did limit the potential interval to 17 protein-coding genes through non-interacting deficiencies. In the course of testing 8 of these potential *Rho1*-interacting genes, we identified a hypomorphic allele of *Rheb* that showed SSNC with *Rho1^E(br)233^* and was close to reaching the threshold for SSNC with *Rho1^E(br)246^* (Table 2). Although we failed to observe an interaction between three additional P-element insertion alleles of *Rheb* and *Rho1^E(br)246^*, we suggest that *Rheb* does interact with *Rho1* during imaginal disc morphogenesis for the following reasons. First, *Rheb^AV4^* behaved similarly in pattern and level of interaction to *Df(3R)Exel6144* with all three *Rho1* alleles tested (Table 2). Second, *zip^E(br)^/+;Rheb^AV4^/+ and RhoGEF2^11-3b^/+;Rheb^AV4^/+* produced 19% and 9% malformed legs, respectively, which although just below the threshold we established for interaction, raises the possibility that *Rheb* interacts with the *Rho* signaling pathway (Table 3). Finally, overexpression of *Rheb* in distal leg segments resulted in a nearly completely penetrant malformed leg phenotype (Figure 3D, E).

An intriguing possibility is that there is an interaction between the Rho1 and Target of rapamycin (Tor) signaling pathways during imaginal disc morphogenesis. We observed an interaction between *Df(2L)Exel7055* and all three tested alleles of *Rho1*. We subsequently limited the *Rho1*-interacting interval to 13 potential genes including *Tor* (Table 2). Although we did not detect an obvious interaction with *Tor*, we consistently observed a trend of ∼5% SSNC between *Tor^ΔP^* and all three *Rho1* alleles (Table 2). In addition, we observed nearly the same level of interaction between another weak P element allele of *Tor* and *Rho1^E(br)246^*. Given the low background in this study, these results stood out even if they did not reach our threshold. In higher eukaryotes, a single TOR protein participates in two functionally distinct protein complexes: TOR complex 1 (TORC1) and TOR complex 2 (TORC2) (reviewed in [48]). TORC1 is a central regulator of cellular growth that integrates a number of growth promoting stimuli including growth factors, low energy and nutrients to regulate ribosome biogenesis and protein synthesis. TORC2 may also regulate cell growth, but importantly also appears to regulate cell morphology by mediating actin cytoskeletal modifications in response to growth factors. Recent evidence indicates that it carries out this function through the activation of Rac and Rho [49]. It is conceivable that TORC2 acts upstream of the Rho1 signaling pathway to regulate the actin cytoskeleton during the cell shape changes and cell rearrangements that drive leg morphogenesis.

An alternative possibility is that Rheb interacts with Rho1 during leg morphogenesis in a pathway that is independent of its role in regulating Tor. Our overexpression studies support this notion. Overexpression of an activated PI3 kinase in distal leg segments results in a highly penetrant growth advantage in those tissues, producing larger distal tibias and tarsal segments with very little overt malformations (Figure 3C). In contrast, overexpressing a wild type *Rheb* transgene gave a consistently strong malformed leg phenotype in addition to the growth advantage (Figure 3D, E). We observed nearly identical results using two independent *Rheb* transgenic lines. In addition, recent evidence suggests that although Rheb has a well-described role as a key upstream activator of TORC1, it may not activate TORC2, and in fact may have an indirect inhibitory effect on TORC2 [50]. Furthermore, clonal analysis of loss of function *Rheb* alleles in *Drosophila* imaginal discs revealed unusual stretched and odd shaped clones that caused the authors of the study to suggest that *Rheb* has other functions in addition to growth control [51]. It will be interesting to determine if the Rheb GTPase has effectors other than Tor and how these potential effortors may intersect with the Rho1 signaling pathway in imaginal disc morphogenesis.

#### Sc2

We identified a single hypomorphic allele of *Sc2*, *Sc2^1^*, which showed SSNC with all three *Rho1* alleles, and also showed a low level interaction with *zip^E(br)^*. Four additional alleles of *Sc2* did not show an interaction with *Rho1^E(br)246^*, raising the possibility that this potential interaction could be due to a second-site mutation on the *Sc2^1^*chromosome. The strongest evidence that this is not the case is that *Sc2* is one of only four genes deleted by two overlapping *Rho1*-interacting deficiencies, and was the only one of these genes to show an interaction with *Rho1*. Specifically, SSNC tests indicated no interaction between *Rho1^E(br)246^* and *ida* or *mge* (Table S2). The final gene in this interval, *Eip63F-1*, is not essential (Vaskova 2000), and is thus less likely to be the *Rho1*-interacting gene. *Sc2* encodes a 302 amino acid protein with two conserved domains, an amino-terminal ubiquitin domain (IPR000626) and a carboxyl-terminal 3-oxo-5-alpha-steroid 4-dehydrogenase domain (IPR001104). This latter domain is highly conserved and catalyzes the terminal step in the microsomal fatty acyl elongation cycle for long chain and very long chain fatty acids (palmitic acid, C16, and larger) [52]. How the synthesis of very long chain fatty acids influences Rho1 signaling is unclear, especially since these enzymes are not required for the synthesis of isoprenoids, and thus not involved in the lipid modification of Rho proteins directly. Rather, it might relate to the overall lipid composition of the plasma membrane. Lipid rafts are specialized membrane microdomains enriched in glycosphingolipids and cholesterol. Numerous membrane proteins involved in signal transduction events, including proteins that regulating Rho signaling, are enriched in these membrane domains [53]. Perhaps the regulated expression of long chain fatty acids via Sc2 modulates lipid raft size and composition, and thereby regulates signal transduction events through Rho1. Additional work will be required to address this hypothesis.

### RhoGEF2 is a key regulator of Rho signaling during leg morphogenesis

One question we were interested in addressing as part of this study is which *RhoGEF* genes were likely functioning during imaginal disc morphogenesis. Our results clearly demonstrate that *RhoGEF2* interacts genetically with *Rho1* in this process (Table 2), supporting previous work from Halsell et al. [23] and Bayer et al. [26]. We found no evidence to definitively support any other potential RhoGEF as interacting with *Rho1* during leg development, although we did identify two deficiencies that uncover putative *RhoGEF* genes that strongly enhance the malformed leg phenotype of *Rho1^E(br)246^* (Table 4). Since there are no loss of function alleles for these genes, we have not been able to address them directly, and thus future studies are needed to test potential roles for *CG30440* and *CG30115* in leg morphogenesis. It should be noted that although we have no evidence to support a role for other *RhoGEF* genes, it is not possible to exclude them as functioning in leg morphogenesis if, for example, they are playing a redundant role or are expressed at levels that render them less dose sensitive.

Since it is clear that RhoGEF2 is functioning with Rho1 during imaginal disc morphogenesis, an important next question is how is RhoGEF2 activated in this process. Previous studies revealed that the subcellular localization of RhoGEF2 shifts to the apical region of cells destined to undergo apical constriction [54], [55]. Since RhoGEF2 has both PH and PDZ domains, its localization may be mediated by the PIP3 level of the plasma membrane or by the expression of a specific protein containing a PDZ binding site. Due to the potential involvement of the Tor signaling pathway in leg morphogenesis, we considered whether PI3 kinase was actively altering the PIP3 content of the plasma membrane in these cells. As an indirect means to assess the PIP3 content of the plasma membrane, we examined prepupal leg imaginal discs from flies expressing a fusion protein containing a PH domain linked to green fluorescent protein (tGPH; [56]). We found no obvious localization of the fusion protein to the plasma membrane in these cells indicating low levels of PIP3 in leg disc cells at these times, although as a control we could detect membrane localization of tGPH in larval salivary glands and fat bodies as expected (data not shown). Therefore these results suggest that RhoGEF2 is localized (and likely activated) through the binding of a specific protein. Perhaps the cloning of *Rho1*-interacting genes from the unidentified intervals may shed light on this issue in the future.

Leg morphogenesis requires a complex interplay of individual cell and collective tissue events, many of which are likely to be regulated by Rho signaling. The identification of three novel *Rho1*-interacting genes and six intervals containing novel *Rho1*-interacting genes, many of which interact genetically with other components of the Rho signaling pathway, provides a great opportunity to define the role of Rho signaling in these distinct morphogenetic events. Since this screen was predicated upon observing defects in leg morphogenesis that persisted until the final adult form of the appendage, it was not possible to determine which of these processes was most affected by the interaction between mutations in *Rho1* and specific deficiencies or mutations. Thus our future challenge is to more accurately define the cellular events occurring during leg morphogenesis such that once we clone the relevant *Rho1*-interacting genes, we can use loss of function clonal analysis and induced RNA interference approaches to determine how these genes function during morphogenesis. In addition, we anticipate that more genetic screens will be performed to identify the *Rho1*-interacting genes playing critical roles at other stages of development such as during gastrulation, segment groove formation and head involution during embryogenesis. In this way we can define the myriad different Rho signaling modules that function during distinct morphogenetic processes during development, and may one day better understand how Rho signaling can be subverted in pathological states including tumor progression and metastasis.

## Supporting Information

Table S1 SSNC results of the primary screen between *Rho1^E(br)246^* and Exelixis deficiencies
(0.47 MB DOC)

Table S2 Secondary screen for all Exelixis deficiencies that showed 10% malformed legs when heterozygous with *Rho1^E(br)246^/+*
(0.30 MB DOC)
