# Supplementary material for: A Second-Site Noncomplementation Screen for Modifiers of Rho1 Signaling during Imaginal Disc Morphogenesis in Drosophila
Source: PLoS One. 2009 Oct 23;4(10):e7574. doi: 10.1371/journal.pone.0007574 (PMC2764050; doi:10.1371/journal.pone.0007574)
Supplement: Table S1 — SSNC results of the primary screen between Rho1E(br)246 and Exelixis deficiencies (0.47 MB DOC) [file pone.0007574.s001.doc]

**Table S1.** SSNC results of the primary screen between *Rho1^E(br)246^* and Exelixis deficiencies

| **Deficiency** | **Cytology^a^** | **% malf. (*n*)^b^** |
| --- | --- | --- |
| *Df(1)Exel6221* | 1B4 ; 1B8 | 4 (75) |
| *Df(1)Exel6223* | 1C4 ; 1D2 | 3 (95) |
| *Df(1)Exel6225* | 1D4 ; 1E3 | 1 (93) |
| *Df(1)Exel6226* | 1E3 ; 1F2 | 1 (85) |
| *Df(1)Exel6227* | 1F3 ; 2B1 | 1 (108) |
| ***Df(1)Exel8196*** | **2B1 ; 2B5** | **9 (78)^c^** |
| *Df(1)Exel6231* | 3A2 ; 3A3 | 4 (96) |
| *Df(1)Exel6233* | 3D2 ; 3D4 | 2 (121) |
| *Df(1)Exel6290* | 4F7 ; 4F10 | 2 (86) |
| *Df(1)Exel6234* | 4F10 ; 5A2 | 5 (105) |
| *Df(1)Exel6235* | 5A2 ; 5A6 | 0 (95) |
| *Df(1)Exel6236* | 5A12 ; 5C2 | 8 (102) |
| *Df(1)Exel6237* | 5C2 ; 5C6 | 4 (82) |
| ***Df(1)Exel6238*** | **5D3 ; 5E4** | **14 (28)** |
| *Df(1)Exel6239* | 5F2 ; 6B1 | 1 (95) |
| *Df(1)Exel6240* | 6B2 ; 6C4 | 0 (95) |
| *Df(1)Exel6241* | 8A2 ; 8B2 | 0 (92) |
| *Df(1)Exel9049* | 8D2 ; 8D3 | 4 (53) |
| *Df(1)Exel6242* | 10C2 ; 10D7 | 1 (96) |
| *Df(1)Exel9050* | 10D5 ; 10D6 | 0 (68) |
| ***Df(1)Exel9053*** | **10D5 ; 10D6** | **10 (70)** |
| *Df(1)Exel6244* | 11A8 ; 11B1 | 2 (60) |
| ***Df(1)Exel6245*** | **11E11 ; 11F4** | **11 (46)** |
| *Df(1)Exel6248* | 12F4 ; 13A1 | 1 (122) |
| *Df(1)Exel6251* | 13E8 ; 13F17 | 0 (86) |
| *Df(1)Exel7463* | 17C2 ; 17D3 | 3 (89) |
| *Df(1)Exel9051* | 17D1 ; 17D3 | 0 (142) |
| *Df(1)Exel9054* | 17D1 ; 17D3 | 0 (121) |
| *Df(1)Exel7464* | 17D1 ; 17E1 | 1 (76) |
| *Df(1)Exel9067* | 17D6 ; 17E1 | 2 (142) |
| *Df(1)Exel6291* | 18A2 ; 18A2 | 3 (88) |
| *Df(1)Exel9068* | 18B4 ; 18B6 | 0 (64) |
| *Df(1)Exel7468* | 18B7 ; 18C8 | 3 (63) |
| ***Df(1)Exel6253*** | **18D13 ; 18F2** | **11 (85)** |
| *Df(1)Exel6254* | 19C4 ; 19D1 | 0 (106) |
| *Df(1)Exel6255* | 20A1 ; 20C1 | 5 (82) |
| *Df(2L)Exel6001* | 21B1 ; 21B1 | 2 (45) |
| *Df(2L)Exel7002* | 21B4 ; 21B7 | 2 (56) |
| *Df(2L)Exel8001* | 21B7 ; 21B8 | 1 (80) |
| *Df(2L)Exel8003* | 21D1 ; 21D2 | 1 (87) |
| *Df(2L)Exel7005* | 21D2 ; 21D4 | 0 (94) |
| *Df(2L)Exel6003* | 21D3 ; 21E3 | 0 (92) |
| *Df(2L)Exel6002* | 21E2 ; 21E2 | 0 (85) |
| *Df(2L)Exel6004* | 21E4 ; 21F1 | 1 (112) |
| *Df(2L)Exel7006* | 21F1 ; 21F4 | 0 (117) |
| *Df(2L)Exel8004* | 21F4 ; 22A3 | 0 (103) |
| *Df(2L)Exel6005* | 22A3 ; 22B1 | 5 (61) |
| *Df(2L)Exel7007* | 22B1 ; 22B5 | 0 (95) |
| *Df(2L)Exel8005* | 22B2 ; 22B8 | 0 (86) |
| *Df(2L)Exel7008* | 22B8 ; 22D1 | 4 (83) |
| *Df(2L)Exel7009* | 22D1 ; 22D5 | 1 (104) |
| *Df(2L)Exel6007* | 22D1 ; 22E1 | 0 (44) |
| *Df(2L)Exel7010* | 22D4 ; 22E1 | 2 (65) |
| *Df(2L)Exel6008* | 22F4 ; 23A3 | 4 (85) |
| *Df(2L)Exel6277* | 23A2 ; 23B1 | 0 (119) |
| *Df(2L)Exel7014* | 23C4 ; 23C5 | 2 (49) |
| *Df(2L)Exel7015* | 23D1 ; 23E3 | 2 (65) |
| *Df(2L)Exel8008* | 23E3 ; 23E5 | 1 (123) |
| *Df(2L)Exel7016* | 23F3 ; 23F3 | 3 (75) |
| *Df(2L)Exel7017* | 23F6 ; 24A2 | 1 (146) |
| *Df(2L)Exel7018* | 24A1 ; 24C2 | 0 (53) |
| *Df(2L)Exel6009* | 24C3 ; 24C8 | 0 (92) |
| *Df(2L)Exel8010* | 24C8 ; 24D4 | 0 (90) |
| *Df(2L)Exel8011* | 24D8 ; 25A1 | 0 (105) |
| *Df(2L)Exel6010* | 25A6 ; 25B1 | 2 (95) |
| *Df(2L)Exel9062* | 25B1 ; 25B1 | 0 (95) |
| *Df(2L)Exel8012* | 25B1 ; 25B5 | 0 (52) |
| *Df(2L)Exel7021* | 25B3 ; 25B9 | 3 (68) |
| *Df(2L)Exel8013* | 25B5 ; 25B10 | 0 (88) |
| *Df(2L)Exel7022* | 25B10 ; 25C3 | 1 (81) |
| *Df(2L)Exel6011* | 25C8 ; 25D5 | 0 (74) |
| *Df(2L)Exel6012* | 25D5 ; 25E6 | 0 (71) |
| *Df(2L)Exel7023* | 25E5 ; 25F1 | 0 (84) |
| *Df(2L)Exel6256* | 25E6 ; 25F2 | 2 (66) |
| *Df(2L)Exel8016* | 25E6 ; 25F2 | 0 (75) |
| *Df(2L)Exel6013* | 25F2 ; 25F5 | 5 (80) |
| *Df(2L)Exel6014* | 25F5 ; 26A3 | 1 (116) |
| *Df(2L)Exel7024* | 26A1 ; 26A8 | 0 (100) |
| *Df(2L)Exel6015* | 26B9 ; 26C1 | 0 (57) |
| *Df(2L)Exel6016* | 26C1 ; 26D1 | 1 (68) |
| *Df(2L)Exel9038* | 26C2 ; 26C3 | 1 (109) |
| *Df(2L)Exel7027* | 26F5 ; 27B1 | 0 (72) |
| *Df(2L)Exel7029* | 27C4 ; 27D4 | 1 (83) |
| *Df(2L)Exel8019* | 27E2 ; 27E4 | 0 (93) |
| ***Df(2L)Exel6017*** | **27E4 ; 27F5** | **19 (54)** |
| *Df(2L)Exel7031* | 27F2 ; 28A3 | 1 (76) |
| *Df(2L)Exel9031* | 28B4 ; 28C1 | 0 (190) |
| *Df(2L)Exel7034* | 28E1 ; 28F1 | 3 (70) |
| *Df(2L)Exel7038* | 29C4 ; 29D5 | 2 (94) |
| *Df(2L)Exel7039* | 29D5 ; 29F1 | 3 (65) |
| ***Df(2L)Exel7040*** | **29F1 ; 29F6** | **10 (52)** |
| *Df(2L)Exel8022* | 30B1 ; 30B4 | 0 (83) |
| *Df(2L)Exel9064* | 30B2 ; 30B3 | 0 (32) |
| *Df(2L)Exel6022* | 30B5 ; 30B11 | 2 (82) |
| *Df(2L)Exel7042* | 30B10 ; 30C1 | 0 (66) |
| *Df(2L)Exel9040* | 30C1 ; 30C1 | 2 (53) |
| *Df(2L)Exel6024* | 30C1 ; 30C9 | 2 (58) |
| *Df(2L)Exel6025* | 30C9 ; 30E1 | 0 (128) |
| *Df(2L)Exel7043* | 30D1 ; 30F1 | 1 (115) |
| *Df(2L)Exel8024* | 31A1 ; 31B1 | 1 (93) |
| *Df(2L)Exel6026* | 31A2 ; 31D7 | 1 (83) |
| *Df(2L)Exel9032* | 31A3 ; 31B1 | 2 (91) |
| *Df(2L)Exel7046* | 31C3 ; 31D9 | 6 (65) |
| *Df(2L)Exel8026* | 31F5 ; 32B3 | 0 (75) |
| *Df(2L)Exel7049* | 32B1 ; 32C1 | 1 (103) |
| *Df(2L)Exel6027* | 32D1 ; 32D5 | 3 (86) |
| *Df(2L)Exel6028* | 32D5 ; 32E4 | 6 (51) |
| *Df(2L)Exel6029* | 32E4 ; 32F2 | 4 (79) |
| *Df(2L)Exel6030* | 33A2 ; 33B3 | 0 (90) |
| *Df(2L)Exel6031* | 33B3 ; 33C2 | 0 (146) |
| *Df(2L)Exel6032* | 33C2 ; 33D4 | 3 (95) |
| *Df(2L)Exel6033* | 33E4 ; 33F2 | 0 (91) |
| *Df(2L)Exel6034* | 33F2 ; 34A1 | 0 (93) |
| *Df(2L)Exel8028* | 34A1 ; 34A2 | 0 (135) |
| ***Df(2L)Exel7055*** | **34A2 ; 34A7** | **15 (65)** |
| *Df(2L)Exel9023* | 34A6 ; 34A7 | 0 (211) |
| *Df(2L)Exel8029* | 34A6 ; 34B2 | 3 (105) |
| *Df(2L)Exel7059* | 34D3 ; 34E1 | 0 (95) |
| *Df(2L)Exel8032* | 34F1 ; 35A3 | 1 (114) |
| *Df(2L)Exel6035* | 35A3 ; 35B2 | 2 (100) |
| *Df(2L)Exel6036* | 35B1 ; 35B2 | 0 (104) |
| *Df(2L)Exel8033* | 35B1 ; 35B8 | 1 (102) |
| *Df(2L)Exel7061* | 35B7 ; 35C1 | 1 (118) |
| *Df(2L)Exel8034* | 35C5 ; 35D2 | 3 (90) |
| *Df(2L)Exel7063* | 35D2 ; 35D4 | 4 (98) |
| *Df(2L)Exel6037* | 35D4 ; 35D6 | 1 (190) |
| *Df(2L)Exel6038* | 35D6 ; 35E2 | 4 (96) |
| *Df(2L)Exel7065* | 35F8 ; 36A3 | 0 (100) |
| *Df(2L)Exel7066* | 36A1 ; 36A12 | 0 (77)5 |
| *Df(2L)Exel6039* | 36A10 ; 36B3 | 4 (77) |
| *Df(2L)Exel7067* | 36A12 ; 36B1 | 0 (72) |
| *Df(2L)Exel8036* | 36B1 ; 36C9 | 0 (63) |
| *Df(2L)Exel7068* | 36C7 ; 36C10 | 2 (96) |
| *Df(2L)Exel9044* | 36C10 ; 36D1 | 1 (84) |
| *Df(2L)Exel7069* | 36C10 ; 36D3 | 2 (121) |
| *Df(2L)Exel9063* | 36E1 ; 36E1 | 0 (56) |
| *Df(2L)Exel7070* | 36E2 ; 36E6 | 2 (101) |
| *Df(2L)Exel8038* | 36E5 ; 36F5 | 0 (81) |
| *Df(2L)Exel9033* | 36F2 ; 36F2 | 1 (95) |
| *Df(2L)Exel6041* | 36F6 ; 37A2 | 0 (97) |
| *Df(2L)Exel7071* | 37A1 ; 37A4 | 5 (96) |
| *Df(2L)Exel7072* | 37A2 ; 37B6 | 2 (62) |
| *Df(2L)Exel7073* | 37B1 ; 37B9 | 1 (95) |
| *Df(2L)Exel8039* | 37B8 ; 37B11 | 1 (148) |
| *Df(2L)Exel6042* | 37B8 ; 37C5 | 2 (110) |
| *Df(2L)Exel8040* | 37C1 ; 37C5 | 0 (133) |
| *Df(2L)Exel6043* | 37C5 ; 37D7 | 1 (98) |
| *Df(2L)Exel7075* | 37D2 ; 37E1 | 0 (93) |
| *Df(2L)Exel8041* | 37D7 ; 37F2 | 0 (106) |
| *Df(2L)Exel6044* | 37F2 ; 38A3 | 0 (48) |
| *Df(2L)Exel6045* | 38A3 ; 38A7 | 1 (101) |
| *Df(2L)Exel7077* | 38A7 ; 38B2 | 2 (99) |
| *Df(2L)Exel6046* | 38C2 ; 38C7 | 5 (21) |
| *Df(2L)Exel7078* | 38C7 ; 38D5 | 2 (103) |
| *Df(2L)Exel7079* | 38E9 ; 38F3 | 0 (101) |
| *Df(2L)Exel7080* | 38F3 ; 39A2 | 1 (143) |
| *Df(2L)Exel6047* | 39A2 ; 39B4 | 4 (83) |
| *Df(2L)Exel9027* | 39A7 ; 39B2 | 0 (155) |
| *Df(2L)Exel6048* | 39B4 ; 39D1 | 0 (103) |
| *Df(2L)Exel7081* | 39D1 ; 39E6 | 0 (56) |
| *Df(2L)Exel7082* | 39E3 ; 40B3 | 1 (104) |
| *Df(2L)Exel6049* | 39E7 ; 40D3 | 1 (149) |
| *Df(2R)Exel6050* | 42C7 ; 42D6 | 0 (146) |
| *Df(2R)Exel6051* | 42D6 ; 42E4 | 0 (93) |
| *Df(2R)Exel6283* | 42E7 ; 43A1 | 2 (185) |
| *Df(2R)Exel7092* | 42F3 ; 43E12 | 5 (76) |
| *Df(2R)Exel6052* | 43D1 ; 42F3 | 3 (130) |
| *Df(2R)Exel6053* | 43D5 ; 43E9 | 4 (126) |
| *Df(2R)Exel6054* | 43E9 ; 43E18 | 5 (134) |
| *Df(2R)Exel6055* | 43F1 ; 44A4 | 2 (132) |
| ***Df(2R)Exel7094*** | **44A4 ; 44B4** | **12 (153)** |
| *Df(2R)Exel6056* | 44A4 ; 44C2 | 5 (100) |
| *Df(2R)Exel7095* | 44B3 ; 44C2 | 2 (154) |
| *Df(2R)Exel6057* | 44B8 ; 44C4 | 4 (96) |
| *Df(2R)Exel6058* | 44C4 ; 44D1 | 3 (72) |
| *Df(2R)Exel7096* | 44C6 ; 44D3 | 8 (136) |
| *Df(2R)Exel8047* | 44D4 ; 44D5 | 1 (151) |
| ***Df(2R)Exel7098*** | **44D5 ; 44E3** | **10 (71)** |
| *Df(2R)Exel8049* | 45F1 ; 46A1 | 0 (100) |
| *Df(2R)Exel7112* | 47B13 ; 47C3 | 0 (77) |
| *Df(2R)Exel6059* | 47C5 ; 47D6 | 0 (101) |
| *Df(2R)Exel6060* | 47D6 ; 47F8 | 9 (56) |
| *Df(2R)Exel6061* | 48F1 ; 49A6 | 5 (86) |
| *Df(2R)Exel7121* | 49B5 ; 49B12 | 4 (80) |
| *Df(2R)Exel8056* | 49C2 ; 49E1 | 2 (53) |
| *Df(2R)Exel7124* | 49D4 ; 50A1 | 0 (65) |
| *Df(2R)Exel7123* | 49D5 ; 49E6 | 2 (104) |
| *Df(2R)Exel6062* | 49E6 ; 49F1 | 0 (112) |
| *Df(2R)Exel8057* | 49F1 ; 49F10 | 3 (58) |
| *Df(2R)Exel7128* | 50C5 ; 50C9 | 4 (74) |
| *Df(2R)Exel7130* | 50D4 ; 50E4 | 0 (58) |
| *Df(2R)Exel7131* | 50E4 ; 50F6 | 3 (61) |
| *Df(2R)Exel8059* | 51A4 ; 51B1 | 0 (63) |
| *Df(2R)Exel6284* | 51B1 ; 51C2 | 2 (166) |
| *Df(2R)Exel7135* | 51E2 ; 51E11 | 0 (34) |
| *Df(2R)Exel9015* | 51F11 ; 51F12 | 2 (60) |
| *Df(2R)Exel6285* | 52A4 ; 52B5 | 3 (151) |
| *Df(2R)Exel9026* | 52A13 ; 52A13 | 4 (70) |
| *Df(2R)Exel7137* | 52B1 ; 52C8 | 0 (53) |
| *Df(2R)Exel7138* | 52D1 ; 52D12 | 5 (41) |
| *Df(2R)Exel7139* | 52D11 ; 52E4 | 0 (54) |
| *Df(2R)Exel9060* | 52E11 ; 52F1 | 2 (112) |
| *Df(2R)Exel6063* | 52F6 ; 53C3 | 0 (108) |
| *Df(2R)Exel7142* | 53B1 ; 53C4 | 5 (64) |
| *Df(2R)Exel6064* | 53C10 ; 53D2 | 2 (88) |
| *Df(2R)Exel6066* | 53C14 ; 54B6 | 3 (118) |
| *Df(2R)Exel7145* | 53D4 ; 53D12 | 0 (102) |
| ***Df(2R)Exel6065*** | **53D14 ; 53F9** | **17 (163)** |
| *Df(2R)Exel7149* | 54C10 ; 54D5 | 0 (91) |
| *Df(2R)Exel7150* | 54E1 ; 54E9 | 0 (109) |
| *Df(2R)Exel7157* | 55E2 ; 55E10 | 0 (109) |
| *Df(2R)Exel7158* | 55E9 ; 55F6 | 3 (124) |
| *Df(2R)Exel6067* | 55F8 ; 55F8 | 2 (113) |
| *Df(2R)Exel6068* | 56A1 ; 56B5 | 5 (140) |
| *Df(2R)Exel6069* | 56B5 ; 56C11 | 0 (107) |
| *Df(2R)Exel7162* | 56F11 ; 56F16 | 3 (87) |
| *Df(2R)Exel7164* | 57A6 ; 57A9 | 0 (114) |
| *Df(2R)Exel6070* | 57A6 ; 57B3 | 0 (215) |
| *Df(2R)Exel6071* | 57B3 ; 57B16 | 1 (181) |
| *Df(2R)Exel7167* | 57B16 ; 57C7 | 1 (72) |
| *Df(2R)Exel6073* | 57B16 ; 57C7 | 0 (187) |
| *Df(2R)Exel6072* | 57B16 ; 57D4 | 2 (186) |
| *Df(2R)Exel6076* | 57D13 ; 57F3 | 1 (180) |
| *Df(2R)Exel6077* | 57F10 ; 58A3 | 1 (140) |
| *Df(2R)Exel7169* | 58A3 ; 58B1 | 0 (128) |
| *Df(2R)Exel6078* | 58B1 ; 57F11 | 3 (187) |
| *Df(2R)Exel7171* | 58C1 ; 58D2 | 0 (114) |
| *Df(2R)Exel7173* | 58D4 ; 58E5 | 1 (78) |
| *Df(2R)Exel6079* | 59A3 ; 59B1 | 3 (201) |
| *Df(2R)Exel7176* | 59B4 ; 59C2 | 3 (107) |
| *Df(2R)Exel7177* | 59C3 ; 59D2 | 0 (102) |
| *Df(2R)Exel7178* | 59D5 ; 59D10 | 1 (89) |
| *Df(2R)Exel7179* | 59D11 ; 59E3 | 0 (104) |
| *Df(2R)Exel7180* | 59E3 ; 59F6 | 0 (115) |
| *Df(2R)Exel7182* | 60A13 ; 60A16 | 1 (154) |
| *Df(2R)Exel9024* | 60A16 ; 60A16 | 0 (53) |
| *Df(2R)Exel6081* | 60B4 ; 60C6 | 1 (150) |
| *Df(2R)Exel9043* | 60C7 ; 60C7 | 1 (121) |
| *Df(2R)Exel7185* | 60C8 ; 60D3 | 0 (109) |
| *Df(3L)Exel6083* | 61A6 ; 61B2 | 2 (124) |
| *Df(3L)Exel6084* | 61B2 ; 61C1 | 6 (242) |
| *Df(3L)Exel9057* | 61C1 ; 61C1 | 1 (77) |
| *Df(3L)Exel6085* | 61C3 ; 61C9 | 1 (122) |
| *Df(3L)Exel6086* | 61C9 ; 61E1 | 0 (208) |
| *Df(3L)Exel6087* | 62A2 ; 62A7 | 3 (370) |
| *Df(3L)Exel6088* | 62B4 ; 62B7 | 6 (281) |
| *Df(3L)Exel6089* | 62D1 ; 62D4 | 4 (381) |
| *Df(3L)Exel6090* | 62E1 ; 62E3 | 1 (142) |
| *Df(3L)Exel6091* | 62E8 ; 62F5 | 1 (135) |
| *Df(3L)Exel6092* | 62F5 ; 63A3 | 1 (161) |
| *Df(3L)Exel6093* | 63C1 ; 63D3 | 4 (138) |
| *Df(3L)Exel6094* | 63D2 ; 63E1 | 3 (104) |
| *Df(3L)Exel6095* | 63E1 ; 63E3 | 2 (127) |
| *Df(3L)Exel6096* | 63E3 ; 63E4 | 1 (134) |
| *Df(3L)Exel6097* | 63E3 ; 63F2 | 4 (112) |
| ***Df(3L)Exel6098*** | **63F2 ; 63F7** | **8 (120)^c^** |
| *Df(3L)Exel6099* | 63F7 ; 64A5 | 2 (157) |
| *Df(3L)Exel9000* | 64A10 ; 64A12 | 3 (196) |
| *Df(3L)Exel8098* | 64A12 ; 64B4 | 1 (163) |
| *Df(3L)Exel9001* | 64B2 ; 64B4 | 0 (220) |
| *Df(3L)Exel6101* | 64B5 ; 64B11 | 4 (349) |
| *Df(3L)Exel9028* | 64B9 ; 64B9 | 2 (121) |
| *Df(3L)Exel9058* | 64B9 ; 64B9 | 2 (372) |
| *Df(3L)Exel7208* | 64B9 ; 64B15 | 7 (192) |
| *Df(3L)Exel6102* | 64B15 ; 64C5 | 2 (114) |
| *Df(3L)Exel6103* | 64C5 ; 64C10 | 2 (336) |
| *Df(3L)Exel6104* | 64C8 ; 64D1 | 4 (268) |
| *Df(3L)Exel6105* | 64D1 ; 64D6 | 2 (58) |
| *Df(3L)Exel6106* | 64D6 ; 64E2 | 4 (116) |
| *Df(3L)Exel6107* | 64E5 ; 64F5 | 0 (109) |
| *Df(3L)Exel7210* | 65A1 ; 65A5 | 0 (138) |
| *Df(3L)Exel8101* | 65A3 ; 65A9 | 0 (175) |
| *Df(3L)Exel6108* | 65A8 ; 65A11 | 1 (90) |
| *Df(3L)Exel6109* | 65C3 ; 65D3 | 4 (101) |
| *Df(3L)Exel6110* | 65E3 ; 65E5 | 0 (107) |
| *Df(3L)Exel8104* | 65F7 ; 66A4 | 0 (137) |
| *Df(3L)Exel6279* | 66A17 ; 66B5 | 2 (154) |
| *Df(3L)Exel9034* | 66A22 ; 66B3 | 2 (162) |
| *Df(3L)Exel6112* | 66B5 ; 66C8 | 2 (112) |
| *Df(3L)Exel6114* | 67B10 ; 67C5 | 0 (133) |
| *Df(3L)Exel9048* | 67D1 ; 67D2 | 1 (226) |
| *Df(3L)Exel6115* | 68E1 ; 68E1 | 2 (127) |
| *Df(3L)Exel6116* | 68F2 ; 69A2 | 2 (124) |
| *Df(3L)Exel6117* | 69D1 ; 69E2 | 1 (108) |
| *Df(3L)Exel6118* | 70A2 ; 70A5 | 0 (56) |
| *Df(3L)Exel9017* | 70B1 ; 70B2 | 2 (298) |
| *Df(3L)Exel6119* | 70B2 ; 70C2 | 0 (99) |
| *Df(3L)Exel6120* | 70D1 ; 70D3 | 4 (106) |
| *Df(3L)Exel6121* | 70D3 ; 70D4 | 3 (148) |
| *Df(3L)Exel6122* | 70D4 ; 70D4 | 0 (122) |
| *Df(3L)Exel6123* | 70D7 ; 70E4 | 0 (108) |
| *Df(3L)Exel6125* | 71A3 ; 71B3 | 1 (109) |
| *Df(3L)Exel6126* | 71A3 ; 71B3 | 2 (164) |
| *Df(3L)Exel6262* | 71B3 ; 71C1 | 1 (141) |
| *Df(3L)Exel6127* | 72D1 ; 72D7 | 2 (144) |
| *Df(3L)Exel6128* | 72D9 ; 72D10 | 3 (118) |
| *Df(3L)Exel6129* | 72F1 ; 73A2 | 1 (147) |
| *Df(3L)Exel6130* | 73B5 ; 73D1 | 3 (61) |
| *Df(3L)Exel9002* | 73D1 ; 73D1 | 2 (164) |
| *Df(3L)Exel9003* | 73D1 ; 73D4 | 1 (161) |
| *Df(3L)Exel9004* | 73D1 ; 73D5 | 0 (98) |
| *Df(3L)Exel7253* | 73D5 ; 73E4 | 1 (162) |
| *Df(3L)Exel6131* | 74A1 ; 74A1 | 1 (124) |
| *Df(3L)Exel6132* | 74B2 ; 74D2 | 2 (151) |
| *Df(3L)Exel9006* | 75A2 ; 75A4 | 2 (108) |
| *Df(3L)Exel6133* | 75B4 ; 75B11 | 2 (132) |
| *Df(3L)Exel6134* | 75C7 ; 75D4 | 1 (128) |
| *Df(3L)Exel9046* | 76A5 ; 76A6 | 1 (141) |
| *Df(3L)Exel9007* | 76B3 ; 76B11 | 2 (108) |
| *Df(3L)Exel9008* | 76B3 ; 76B11 | 4 (112) |
| *Df(3L)Exel9009* | 76B5 ; 76B11 | 1 (153) |
| *Df(3L)Exel9011* | 76B8 ; 76B11 | 0 (143) |
| *Df(3L)Exel6135* | 76B11 ; 76C4 | 5 (281) |
| *Df(3L)Exel9061* | 76C2 ; 76C3 | 0 (206) |
| *Df(3L)Exel9045* | 76D1 ; 76D2 | 1 (158) |
| *Df(3L)Exel6136* | 77B2 ; 77C6 | 1 (142) |
| *Df(3L)Exel9065* | 78D5 ; 78D4 | 2 (164) |
| *Df(3L)Exel9066* | 78D5 ; 78D6 | 4 (110) |
| *Df(3L)Exel6137* | 78F4 ; 79A4 | 0 (136) |
| *Df(3L)Exel6138* | 79D3 ; 79E3 | 2 (103) |
| *Df(3R)Exel6140* | 82A1 ; 82A4 | 1 (133) |
| *Df(3R)Exel6141* | 82B2 ; 82C3 | 4 (168) |
| *Df(3R)Exel6142* | 82D2 ; 82D6 | 0 (123) |
| *Df(3R)Exel6143* | 82E4 ; 82E8 | 0 (137) |
| *Df(3R)Exel9029* | 83A1 ; 83A3 | 1 (91) |
| ***Df(3R)Exel6144*** | **83A6 ; 83B6** | **10 (249)** |
| *Df(3R)Exel7283* | 83B7 ; 83C2 | 2 (233) |
| *Df(3R)Exel6145* | 83C1 ; 83C4 | 2 (172) |
| *Df(3R)Exel7284* | 83C4 ; 83D2 | 0 (212) |
| *Df(3R)Exel6146* | 84C8 ; 84D9 | 1 (142) |
| *Df(3R)Exel6263* | 84E6 ; 84E13 | 0 (112) |
| *Df(3R)Exel6147* | 84F6 ; 84F13 | 2 (142) |
| *Df(3R)Exel6148* | 84F12 ; 85A2 | 2 (130) |
| *Df(3R)Exel6149* | 85A2 ; 85A2 | 1 (145) |
| *Df(3R)Exel8143* | 85A5 ; 85B2 | 1 (171) |
| *Df(3R)Exel6150* | 85A5 ; 85B6 | 2 (152) |
| *Df(3R)Exel6151* | 85C3 ; 85C11 | 2 (142) |
| *Df(3R)Exel6152* | 85C11 ; 85D2 | 1 (160) |
| *Df(3R)Exel9036* | 85D11 ; 85D11 | 1 (166) |
| *Df(3R)Exel6153* | 85D21 ; 85E1 | 0 (145) |
| *Df(3R)Exel6264* | 85D24 ; 85E5 | 1 (138) |
| *Df(3R)Exel6154* | 85E9 ; 85F1 | 2 (155) |
| *Df(3R)Exel6155* | 85F1 ; 85F10 | 2 (145) |
| *Df(3R)Exel6265* | 85F10 ; 85F16 | 4 (111) |
| *Df(3R)Exel6156* | 85F16 ; 86B1 | 1 (140) |
| *Df(3R)Exel6157* | 86B1 ; 86B3 | 0 (93) |
| *Df(3R)Exel6158* | 86C2 ; 86C3 | 0 (138) |
| *Df(3R)Exel6159* | 86C3 ; 86C7 | 0 (96) |
| *Df(3R)Exel7305* | 86C6 ; 86C7 | 0 (145) |
| *Df(3R)Exel7306* | 86C7 ; 86D5 | 1 (178) |
| *Df(3R)Exel8152* | 86D7 ; 86D9 | 3 (118) |
| *Df(3R)Exel7308* | 86D9 ; 86D9 | 1 (168) |
| *Df(3R)Exel9018* | 86E2 ; 86E4 | 3 (148) |
| *Df(3R)Exel6160* | 86E4 ; 86E11 | 0 (141) |
| *Df(3R)Exel8153* | 86E8 ; 86E14 | 4 (128) |
| *Df(3R)Exel6276* | 86E11 ; 86E11 | 1 (112) |
| *Df(3R)Exel7309* | 86E13 ; 86E16 | 1 (133) |
| *Df(3R)Exel8154* | 86E13 ; 86E18 | 4 (104) |
| *Df(3R)Exel6161* | 86E14 ; 86E18 | 0 (141) |
| *Df(3R)Exel9019* | 86E17 ; 86E18 | 1 (138) |
| *Df(3R)Exel7310* | 86E18 ; 87A1 | 6 (232) |
| *Df(3R)Exel6163* | 87A1 ; 87A4 | 1 (128) |
| *Df(3R)Exel6162* | 87A1 ; 87B5 | 1 (120) |
| *Df(3R)Exel7312* | 87A4 ; 87A7 | 4 (114) |
| *Df(3R)Exel8155* | 87A4 ; 87A9 | 2 (113) |
| *Df(3R)Exel7313* | 87A9 ; 87B5 | 0 (128) |
| *Df(3R)Exel7314* | 87B3 ; 87B8 | 1 (253) |
| *Df(3R)Exel6164* | 87B5 ; 87B10 | 1 (152) |
| *Df(3R)Exel6165* | 87B5 ; 87B10 | 3 (142) |
| *Df(3R)Exel7315* | 87B8 ; 87B9 | 1 (147) |
| *Df(3R)Exel7316* | 87B9 ; 87B11 | 0 (124) |
| *Df(3R)Exel7317* | 87B10 ; 87C3 | 1 (123) |
| *Df(3R)Exel6166* | 87C5 ; 87C7 | 1 (151) |
| *Df(3R)Exel7318* | 87C7 ; 87D5 | 1 (95) |
| *Df(3R)Exel8157* | 87D8 ; 87D10 | 0 (103) |
| *Df(3R)Exel6167* | 87D10 ; 87E3 | 2 (100) |
| *Df(3R)Exel8158* | 87E3 ; 87E8 | 4 (117) |
| *Df(3R)Exel6168* | 87E3 ; 87E8 | 0 (156) |
| *Df(3R)Exel7320* | 87E8 ; 87F2 | 0 (157) |
| *Df(3R)Exel6169* | 87F2 ; 87F10 | 0 (69) |
| *Df(3R)Exel6170* | 87F10 ; 87F14 | 0 (132) |
| *Df(3R)Exel6288* | 87F14 ; 88A4 | 0 (93) |
| *Df(3R)Exel6171* | 87F14 ; 88A4 | 1 (68) |
| *Df(3R)Exel8159* | 88A4 ; 88B1 | 1 (124) |
| *Df(3R)Exel7321* | 88A9 ; 88B1 | 0 (141) |
| *Df(3R)Exel6267* | 88B1 ; 88C2 | 0 (121) |
| *Df(3R)Exel8160* | 88C10 ; 88D6 | 1 (125) |
| *Df(3R)Exel6275* | 88D1 ; 88D7 | 2 (120) |
| *Df(3R)Exel6172* | 88D5 ; 88D7 | 1 (165) |
| *Df(3R)Exel6173* | 88D7 ; 88E1 | 2 (154) |
| *Df(3R)Exel6174* | 88F1 ; 88F7 | 1 (140) |
| *Df(3R)Exel7326* | 88F7 ; 89A5 | 3 (116) |
| *Df(3R)Exel8162* | 89A5 ; 89A8 | 0 (185) |
| *Df(3R)Exel7327* | 89A8 ; 89B1 | 3 (95) |
| ***Df(3R)Exel7328*** | **89B1 ; 89B9** | **8 (227)^c^** |
| *Df(3R)Exel7330* | 89B13 ; 89B17 | 0 (148) |
| *Df(3R)Exel7329* | 89B14 ; 89B19 | 1 (120) |
| *Df(3R)Exel9055* | 89B16 ; 89B17 | 4 (216) |
| *Df(3R)Exel6269* | 89B17 ; 89B18 | 4 (109) |
| *Df(3R)Exel8163* | 89B17 ; 89B18 | 0 (156( |
| *Df(3R)Exel6270* | 89D2 ; 89D8 | 1 (138) |
| *Df(3R)Exel8165* | 89E8 ; 89E11 | 2 (165) |
| *Df(3R)Exel6176* | 89E11 ; 89F1 | 0 (129) |
| ***Df(3R)Exel6178*** | **90E7 ; 91A5** | **18 (212)** |
| ***Df(3R)Exel6179*** | **91A5 ; 91B5** | **12 (272)** |
| *Df(3R)Exel9030* | 91B5 ; 91B6 | 1 (135) |
| *Df(3R)Exel6180* | 91B5 ; 91C5 | 8 (265) |
| *Df(3R)Exel6181* | 91C5 ; 91D5 | 2 (129) |
| *Df(3R)Exel6182* | 91D5 ; 91E4 | 4 (267) |
| *Df(3R)Exel6183* | 91E4 ; 91F4 | 7 (261) |
| *Df(3R)Exel6184* | 92A5 ; 92A11 | 0 (143) |
| *Df(3R)Exel6185* | 92E2 ; 92F1 | 1 (104) |
| ***Df(3R)Exel6272*** | **93A7 ; 93B13** | **11 (83)** |
| *Df(3R)Exel6186* | 93E9 ; 93E9 | 4 (281) |
| *Df(3R)Exel6187* | 93F6 ; 93F14 | 2 (171) |
| *Df(3R)Exel6188* | 93F14 ; 94A1 | 9 (136) |
| *Df(3R)Exel6189* | 93F14 ; 94A2 | 2 (188) |
| *Df(3R)Exel6190* | 94A2 ; 94A9 | 0 (233) |
| *Df(3R)Exel6191* | 94A9 ; 94B2 | 2 (178) |
| *Df(3R)Exel6273* | 94B2 ; 94B11 | 4 (138) |
| *Df(3R)Exel6192* | 94B11 ; 94D3 | 2 (126) |
| *Df(3R)Exel6193* | 94D3 ; 94E4 | 2 (123) |
| *Df(3R)Exel6274* | 94E4 ; 94E11 | 7 (215) |
| *Df(3R)Exel6280* | 94E5 ; 94E11 | 3 (160) |
| *Df(3R)Exel9012* | 94E9 ; 94E13 | 2 (175) |
| *Df(3R)Exel6194* | 94F1 ; 95A4 | 2 (159) |
| *Df(3R)Exel6195* | 95A4 ; 95B1 | 5 (324) |
| *Df(3R)Exel9013* | 95B1 ; 95B5 | 5 (119) |
| *Df(3R)Exel9014* | 95B1 ; 95D1 | 6 (162) |
| *Df(3R)Exel6196* | 95C12 ; 95D8 | 4 (136) |
| *Df(3R)Exel6197* | 95D8 ; 95E5 | 7 (254) |
| *Df(3R)Exel6198* | 95E1 ; 95F8 | 2 (96) |
| *Df(3R)Exel6199* | 95F8 ; 96A2 | 0 (149) |
| *Df(3R)Exel8178* | 95F8 ; 96A6 | 1 (144) |
| *Df(3R)Exel7357* | 96A2 ; 96A13 | 1 (100) |
| *Df(3R)Exel6200* | 96A20 ; 96B4 | 2 (159) |
| *Df(3R)Exel6201* | 96C2 ; 96C3 | 3 (173) |
| *Df(3R)Exel9056* | 96C4 ; 96C5 | 1 (177) |
| *Df(3R)Exel6202* | 96D1 ; 96D1 | 2 (200) |
| *Df(3R)Exel6203* | 96E2 ; 96E6 | 0 (168) |
| *Df(3R)Exel6204* | 96F9 ; 97A6 | 4 (131) |
| *Df(3R)Exel6205* | 97D12 ; 97E1 | 0 (180) |
| *Df(3R)Exel6206* | 97E1 ; 97E5 | 0 (171) |
| *Df(3R)Exel6208* | 97E5 ; 97E11 | 0 (112) |
| *Df(3R)Exel6259* | 98C4 ; 98D6 | 1 (147) |
| *Df(3R)Exel6209* | 98D6 ; 98E1 | 3 (129) |
| *Df(3R)Exel6210* | 98E1 ; 98F5 | 0 (107) |
| *Df(3R)Exel6211* | 98F5 ; 98F6 | 1 (141) |
| *Df(3R)Exel6212* | 99A1 ; 99A5 | 2 (157) |
| *Df(3R)Exel9025* | 99B10 ; 99B10 | 1 (150) |
| *Df(3R)Exel6213* | 99C5 ; 99D1 | 7 (276) |
| *Df(3R)Exel6214* | 99D5 ; 99E2 | 4 (81) |
| *Df(3R)Exel6216* | 99F6 ; 99F7 | 1 (144) |
| *Df(3R)Exel6215* | 99F6 ; 99F8 | 4 (121) |
| *Df(3R)Exel7378* | 99F8 ; 100A5 | 4 (140) |
| ***Df(3R)Exel9020*** | **100A4 ; 100A5** | **10 (241)** |
| *Df(3R)Exel8194* | 100A4 ; 100A7 | 4 (102) |
| *Df(3R)Exel6217* | 100A6 ; 100A7 | 1 (163) |
| *Df(3R)Exel7379* | 100B2 ; 100B3 | 4 (93) |
| *Df(3R)Exel6219* | 100B4 ; 100B8 | 4 (105) |
| *Df(3R)Exel6218* | 100B5 ; 100C1 | 1 (175) |

^a^Cytology is taken from (Parks AL, Cook KR, Belvin M, Dompe NA, Fawcett R, et al. 2004. Systematic generation of high-resolution deletion coverage of the *Drosophila melanogaster* genome. Nat Genet 36: 288-392.) which reflects release 3 of the *Drosophila* genome, except for the *Rho1*-interacting deficiencies (indicated in bold type) which are based upon flybase annotations as of January 2009 (reflects release 5 of the *Drosophila* genome). ^b^% malformed indicates the percentage of animals heterozygous for *Rho1^E(br)246^* and heterozygous for the indicated Exelixis deficiency showing the malformed leg phenotype in at least one leg. *n*, total number of flies of the indicated genotype that were scored. ^c^Interactions showing less than 10% malformed legs overall that had individual vials showing greater than 10% malformations and were thus subjected to secondary screening (Table 2 and Supplemental Table 2).
