# Supplementary material for: A Second-Site Noncomplementation Screen for Modifiers of Rho1 Signaling during Imaginal Disc Morphogenesis in Drosophila
Source: PLoS One. 2009 Oct 23;4(10):e7574. doi: 10.1371/journal.pone.0007574 (PMC2764050; doi:10.1371/journal.pone.0007574)
Supplement: Table S2 — Secondary screen for all Exelixis deficiencies that showed 10% malformed legs when heterozygous with Rho1E(br)246/+ (0.30 MB DOC) [file pone.0007574.s002.doc]

**Table S2.** Secondary screen for all Exelixis deficiencies that showed 10% malformed legs when heterozygous with *Rho1^E(br)246^/+*

|  |  |  | **% malformed legs with *Rho1^*^*(*n*)^c^** | | |
| --- | --- | --- | --- | --- | --- |
| **1° deficiency stock^a^** | **2° deficiency stock or allele** | **Cytology^b^** | ***Rho1^E(br)246^*** | ***Rho1^E(br)233^*** | ***Rho1^E3.10^*** |
| *Df(1)Exel8196* |  | 2B1; 2B5 | 9 (78) | 31 (42) | 50 (28) |
|  | *Df(1)A94* | 1E3 ; 2B12 | 48 (23) |  |  |
|  | *P{lacW}G0129^G0129^* | 2B1 | 0 (39) |  |  |
|  | *br^5^* | 2B3-5 | 9 (33)^d^ | 8 (49)^d^ |  |
|  | *br^1^* | 2B3-5 | 18 (57)^d^ | 21 (38)^d^ | 62 (53)^d^ |
|  | *dor^8^* | 2B5 | 11 (61) |  |  |
| *Df(1)Exel6238* |  | 5D3 ; 5E4 | 14 (28) | 33 (37) | 14 (42) |
|  | *Df(1)ED418* | 5C7 ; 5E4 | 5 (104) |  |  |
|  | *Df(1)5D* | 5D1 ; 5E | 3 (71) |  |  |
|  | *P{lacW}Mipp2^G0303^* | 5D4 | 3 (66) |  |  |
|  | *P{lacW}Mipp2^G0050^* | 5D4 | 1 (80) |  |  |
|  | *P{EPgy2}sqh^EY09875^* | 5E1 | 2 (96) | 2 (85) | 1 (92) |
|  | *sqh^AX3^* | 5E1 | 1 (84) | 0 (144) |  |
|  | *P{lacW}Lag1^G0061^* | 5E4 | 0 (43) |  |  |
|  | *P{lacW}Lag1^G0365^* | 5E4 | 5 (65) |  |  |
|  | *P{lacW}Spt6^G0063^* | 5E4 | 0 (108) |  |  |
| *Df(1)Exel9053* |  | 10D5 ; 10D6 | 10 (70) | 0 (18) | 4 (75) |
| *Df(1)Exel6245* |  | 11E11 ; 11F4 | 11 (46) | 11 (47) | 0 (84) |
|  | *Df(1)N12* | 11D1 ; 11F2 | 13 (82) |  |  |
|  | *Df(1)C246* | 11D1 ; 12A1 | 13 (38) |  |  |
| *Df(1)Exel6253* |  | 18D13 ; 18F2 | 11 (85) | 25 (51) | 18 (105) |
|  | *Cdc42^1^* | 18E1 | 52 (40) | 47 (34) | 47 (38) |
|  | *Cdc42^3^* | 18E1 | 9 (99) | 13 (68) | 27 (30) |
|  | *Mer^4^* | 18E1 | 3 (87) |  |  |
|  | *P{lacW}dome^G0282^* | 18E1 | 0 (92) |  |  |
|  | *P{lacW}dome^G0218^* | 18E1 | 0 (82) |  |  |
|  | *P{EPgy2}CG14234^EY01200^* | 18E5 | 3 (117) |  |  |
|  | *PBac{WH}CG14235^f04041^* | 18E5 | 1 (70) |  |  |
|  | *P{lacW}l(1)G0120^G0120^* | 18F2 | 2 (61) |  |  |
| *Df(2L)Exel6017* |  | 27E4 ; 27F5 | 19 (54) | 3 (124) | 1 (107) |
|  | *Df(2L)spd^j2^* | 27B2 ; 27F2 | 13 (122) |  |  |
|  | *Df(2L)ED489* | 27E4 ; 28B1 | 12 (78) |  |  |
|  | *P{lacW}Coprox^k10617^* | 27C7 | 5 (129) | 5 (123) | 1 (147) |
|  | *Wnt4^C1^* | 27E7-8 | 2 (126) |  |  |
|  | *P{EPgy2}Wnt4^EY06939^* | 27E7-8 | 4 (126) |  |  |
|  | *P{EPgy2}CG5261^EY03970^* | 27F4 | 8 (119) |  |  |
|  | *P{EPgy2}CG5261^EY12882^* | 27F4 | 0 (147) |  |  |
| *Df(2L)Exel7040* |  | 29F1 ; 29F6 | 10 (52) |  | 5 (60) |
|  | *Df(2L)N22-14* | 29C1 ; 30C9 | 0 (88) |  |  |
| *Df(2L)Exel7055* |  | 34A2 ; 34A7 | 15 (65) | 10 (52) | 11 (105) |
|  | *Df(2L)prd1.7* | 33B3 ; 34A2 | 0 (172) |  |  |
|  | *Df(2L)ED776,* | 33E4 ; 34A3 | 1 (136) |  |  |
|  | *Df(2L)ED777* | 33E7 ; 34A3 | 0 (72) |  |  |
|  | *Df(2L)ED773* | 33E9 ; 34A3 | 0 (177) |  |  |
|  | *Df(2L)ED778* | 33E9 ; 34A7 | 18 (89) |  |  |
|  | *Df(2L)Exel8028* | 34A1 ; 34A2 | 0 (135) |  |  |
|  | *Df(2L)ED774* | 34A3 ; 34A3 | 0 (121) |  |  |
|  | *Df(2L)BSC30* | 34A3 ; 34B9 | 15 (89) |  |  |
|  | *Df(2L)ED784* | 34A4 ; 34B6 | 32 (31) |  |  |
|  | *Df(2L)Exel9023* | 34A6 ; 34A7 | 0 (211) |  |  |
|  | *P{lacW}Vha68-2^s4214^* | 34A3 | 0 (113) |  |  |
|  | *P{PZ}Vha68-2^01510^* | 34A3 | 2 (127) |  |  |
|  | *P{EP}Vha68-2^EP2364^* | 34A3 | 2 (126) |  |  |
|  | *P{EPgy2}Vha68-1^EY02923^* | 34A4 | 5 (74) |  |  |
|  | *P{EPgy2}A16^EY14223^* | 34A4 | 1 (145) |  |  |
|  | *PBac{RB}A16^e00533^* | 34A4 | 1 (191) |  |  |
|  | *Tor^ΔP^* | 34A4 | 5 (354) | 5 (195) | 5 (129) |
|  | *P{lacW}Tor^k17004^* | 34A4 | 4 (161) |  |  |
|  | *PBac{PB}CG9932^c00144^* | 34A4 | 5 (116) |  |  |
|  | *P{EPgy2}EY12357* | 34A6 | 0 (163) |  |  |
|  | *P{PZ}Sir2^05327a^* | 34A7 | 1 (100) |  |  |
|  | *P{EP}Sir2^EP2300^* | 34A7 | 0 (124) |  |  |
|  | *P{EP}Sir2^EP2384^* | 34A7 | 2 (125) |  |  |
|  | *P{SUPor-P}Sir2^KG00871^* | 34A7 | 3 (157) |  |  |
|  | *P{SUPor-P}Edem2^KG00637^* | 34A8 | 1 (164) |  |  |
|  | *P{EPgy2}CG5439^EY03606^* | 34A8 | 1 (142) |  |  |
|  | *P{EPgy2}CG16974^EY01543^* | 34A8 | 5 (86) |  |  |
|  | *P{EPgy2}CG16972^EY01142^* | 34A9 | 1 (120) |  |  |
| *Df(2R)Exel7094* |  | 44A4 ; 44B4 | 12 (153) | 0 (89) | 4 (49) |
|  | *Df(2R)Exel6055* | 43F1 ; 44A4 | 2 (132) |  |  |
|  | *Df(2R)Exel6056* | 44A4 ; 44C2 | 4 (164) | 0 (74) | 9 (58) |
|  | *Df(2R)Exel7095* | 44B3 ; 44C2 | 1 (274) | 10 (64) | 1 (105) |
| *Df(2R)Exel7098* |  | 44D5 ; 44E3 | 10 (71) | 14 (125) | 9 (183) |
|  | *Df(2R)ED1742* | 44B9 ; 44E3 | 11 (149) |  |  |
|  | *Df(2R)H3D3* | 44D1 ; 44F5 | 8 (77) |  |  |
|  | *Df(2R)ED1770* | 44D8 ; 45B4 | 55 (60) | 60 (58) | 23 (64) |
|  | *Vps25^1^* | 44D5 | 2 (118) |  |  |
|  | *P{lacW}Vps25^k08904^* | 44D5 | 1 (139) |  |  |
|  | *P{EPgy2}CG14749^EY00562^* | 44D5 | 2 (185) |  |  |
|  | *l(2)44DEa^1^* | 44E1-2 | 4 (70) |  |  |
|  | *P{PZ}l(2)44DEa^05847^* | 44E1 | 3 (140) |  |  |
|  | *P{EPgy2}CG14767^EY09017^* | 44E3 | 3 (186) |  |  |
|  | *stmA^cmp44E-1^* | 44E3 | 0 (132) |  |  |
| *Df(2R)Exel6065* |  | 53D14 ; 53F9 | 17 (163) | 34 (127) | 50 (70) |
|  | *Df(2R)ED2751,* | 53D14 ; 53F9 | 38 (128) |  |  |
|  | *Df(2R)ED1* | 53E4 ; 53F9 | 31 (80) |  |  |
|  | *PBac{RB}RhoGEF2^e03784^* | 53E4-F1 | 57 (28) |  |  |
|  | *P{EPgy2}RhoGEF2^EY08391^* | 53E4-F1 | 17 (92) |  |  |
|  | *RhoGEF2^11-3b^* | 53E4-F1 | 37 (252) |  |  |
|  | *P{lacW}tef^k15914^ fat-spondin^k15914^* | 53F2 | 1 (146) |  |  |
|  | *P{lacW}GstS1^k08805^* | 53F7-8 | 2 (46) |  |  |
|  | *P{lacW}GstS1^k09854^* | 53F7-8 | 1 (85) |  |  |
|  | *P{PZ}GstS1^06253^* | 53F7-8 | 0 (66) |  |  |
|  | *P{lacW}GstS1^k11301^* | 53F7-8 | 2 (86) |  |  |
| *Df(3L)Exel6098* |  | 63F2 ; 63F7 | 8 (120) | 8 (76) | 10 (72) |
|  | *Df(3L)ED208* | 63C1 ; 63F5 | 23 (59) | 35 (62) | 56 (19) |
|  | *Df(3L)GN19* | 63F3 ; 64B2 | 1 (111) |  |  |
|  | *Df(3L)ED4341* | 63F6 ; 64B9 | 10 (63) |  |  |
|  | *P{EPgy2}CG17737^EY02210^* | 63C1 | 1 (133) |  |  |
|  | *P{EP}dro6^EP3407^* | 63D1 | 3 (111) |  |  |
|  | *P{PZ}kst^01318^* | 63D2 | 1 (135) |  |  |
|  | *P{XP}kst^d11183^* | 63D2 | 0 (199) |  |  |
|  | *P{EP}YT521-B^EP3725^* | 63D2 | 5 (172) |  |  |
|  | *sty^Δ5^* | 63D2-3 | 2 (119) |  |  |
|  | *sty^226^* | 63D2-3 | 0 (74) |  |  |
|  | *P{lacW}L3659* | 63E1 | 0 (91) |  |  |
|  | *P{lacW}armi^1^* | 63E1 | 2 (198) |  |  |
|  | *P{SUPor-P}armi^KG04664^* | 63E1 | 3 (131) |  |  |
|  | *armi^72.1^* | 63E1 | 2 (165) |  |  |
|  | *P{PZ}eIF5B^09143^* | 63E1 | 1 (143) |  |  |
|  | *P{SUPor-P}eIF5B^KG09489^* | 63E1 | 3 (183) |  |  |
|  | *enc^R1^* | 63F1-3 | 5 (154) |  |  |
|  | *PBac{5HPw+}Eip63E^A310^* | 63E4 | 0 (138) |  |  |
|  | *P{EPgy2}Scsα^EY01819^* | 63F1 | 5 (94) |  |  |
|  | *PBac{PB}Awh^c05541^* | 63F4 | 1 (140) |  |  |
|  | *Awh^63Ea-1^* | 63F4 | 3 (145) |  |  |
|  | *Awh^63Ea-G14^* | 63F4 | 1 (142) |  |  |
|  | *Sc2^1^* | 63F5 | 14 (133) | 33 (131) | 11 (80) |
|  | *P{PZ}Sc2^05634^* | 63F5 | 3 (139) |  |  |
|  | *Sc2^A4^* | 63F5 | 4 (92) |  |  |
|  | *Sc2^F9^* | 63F5 | 0 (95) |  |  |
|  | *P{UASp-YFP.Rab8.Q67L}Sc2^10^* | 63F5 | 0 (126) |  |  |
|  | *ida^B4^* | 63F6 | 3 (79) |  |  |
|  | *ida^D14^* | 63F6 | 0 (44) |  |  |
|  | *mge^A1^* | 63F6 | 0 (121) |  |  |
|  | *mge^B10^* | 63F6 | 0 (99) |  |  |
| *Df(3R)Exel6144* |  | 83A6 ; 83B6 | 10 (249) | 25 (87) | 5 (111) |
|  | *Df(3R)2-2* | 81F4 ; 83A | 4 (148) |  |  |
|  | *Df(3R)ED5177* | 83B4 ; 83B6 | 0 (61) |  |  |
|  | *P{wHy}CG31547^DG06112^* | 83A6 | 2 (129) |  |  |
|  | *P{PZ}Itp-r83A^05616^* | 83A7 | 1 (127) |  |  |
|  | *P{EPgy2}Itp-r83A^EY02522^* | 83A7 | 2 (123) |  |  |
|  | *PBac{5HPw+}mia^B560^* | 83B1 | 2 (131) |  |  |
|  | *Snm1^ZIII-2589^* | 83B1-2 | 9 (94) |  |  |
|  | *Snm1^ZIII-4709^* | 83B1-2 | 3 (203) |  |  |
|  | *CRMP^supI2^* | 83B2 | 1 (170) |  |  |
|  | *P{Mae-UAS.6.11}Rheb^AV4^* | 83B2 | 8 (130) | 14 (77) | 4 (113) |
|  | *P{SUPor-P}Rheb^KG02006^* | 83B2 | 3 (149) |  |  |
|  | *RhebP{EPgy2}Rheb^EY08085^* | 83B2 | 2 (133) |  |  |
|  | *P{Mae-UAS.6.11}Rheb^LA01053^* | 83B2 | 4 (162) |  |  |
|  | *Vha26^j3E7^* | 83B4 | 6 (140) | 10 (100) | 3 (117) |
|  | *P{PZ}exba^03022^* | 83B4 | 2 (126) |  |  |
|  | *PBac{WH}noi^f05442^* | 83B4 | 2 (125) |  |  |
| *Df(3R)Exel7328* |  | 89B1 ; 89B9 | 8 (227) | 37 (84) | 55 (116) |
|  | *Df(3R)Exel7327* | 89A8 ; 89B3 | 3 (95) |  |  |
|  | *Df(3R)bxd100* | 89B6 ; 89E2 | 18 (89) |  |  |
|  | *sbd^E(br)536^* | 89B4-6 |  |  | 14 (72)^d^ |
| *Df(3R)Exel6178* |  | 90E7 ; 91A5 | 18 (212) | 42 (85) | 18 (152) |
|  | *Df(3R)P14* | 90C2 ; 91B2 | 9 (68) | 6 (82) | 14 (59) |
|  | *Df(3R)Cha7* | 90F1 ; 91F5 | 11 (76) |  |  |
|  | *Df(3R)ED5815* | 90F4 ; 91B8 | 8 (75) |  |  |
|  | *sr^461^* | 90E4-F1 | 3 (155) |  |  |
|  | *P{PZ}sr^03999^* | 90E4-F1 | 2 (159) |  |  |
|  | *P{lacW}MED17^s2956^* | 90F6 | 1 (137) |  |  |
|  | *P{SUPor-P}Ssdp^KG03600^* | 90F6 | 1 (295) |  |  |
|  | *P{EPgy2}Ssdp^EY10984^* | 90F6 | 1 (158) |  |  |
|  | *P{EPgy2}CG7998^EY01940^* | 90F8 | 2 (101) |  |  |
|  | *P{GAL4}repo* | 90F9-10 | 0 (128) |  |  |
|  | *P{PZ}repo^03702^* | 90F9-10 | 0 (73) |  |  |
|  | *P{EP}14-3-3ε^EP3578^* | 90F10 | 2 (164) |  |  |
|  | *14-3-3ε^S-696^* | 90F10 | 3 (116) |  |  |
|  | *P{lacW}14-3-3ε^j2B10^* | 90F10 | 2 (192) |  |  |
|  | *PBac{PB}eIF-1A^c04533^* | 91A1 | 1 (133) |  |  |
|  | *P{EP}eIF-1A^EP935^* | 91A1 | 2 198) |  |  |
|  | *PBac{PB}CG8064^c05886^* | 91A1 | 0 (73) |  |  |
|  | *P{PZ}Dlc90F^05090b^* | 91A2 | 0 (109) |  |  |
|  | *P{PZ}Dlc90F^04091^* | 91A2 | 0 (87) |  |  |
|  | *P{PZ}Dlc90F^05089^* | 91A2 | 1 (89) |  |  |
|  | *P{SUPor-P}Dlc90F^KG06855^* | 91A2 | 0 (134) |  |  |
| *Df(3R)Exel6179* |  | 91A5 ; 91B5 | 12 (272) | 21 (112) | 11 (159) |
|  | *Df(3R)Cha1a* | 91A2 ; 92A1 | 28 (46) |  |  |
|  | *Df(3R)ED2* | 91A5 ; 91F1 | 15 (127) | 28 (25) |  |
|  | *Df(3R)BX5* | 91B1 ; 91D2 | 4 (134) |  |  |
|  | *Df(3R)07280* | 91B2 ; 91C1 | 13 (125) |  |  |
|  | *PBac{3HPy+}cona^C061^* | 91A5 | 1 (105) |  |  |
|  | *P{PZ}sprd^05284^ koko^05284^ Vha100-2^05284^* | 91A5 | 0 (133) |  |  |
|  | *P{PZ}fray^07551^* | 91B5 | 1 (172) |  |  |
| *Df(3R)Exel6272* |  | 93A7 ; 93B13 | 11 (83) | 9 (57) | 3 (80) |
|  | *Df(3R)BSC43* | 92F7 ; 93B6 | 0 (41) |  |  |
|  | *Df(3R)e-N19* | 93B2 ; 94A8 | 5 (63) |  |  |
| *Df(3R)Exel9020* |  | 100A4 ; 100A5 | 10 (241) | 14 (111) | 10 (167) |
|  | *Df(3R)tll-e* | 100A2 ; 100C3 | 0 (68) |  |  |
|  | *Df(3R)Exel6217* | 100A6 ; 100A7 | 0 (110) |  |  |
|  | *Df(3R)ED6346* | 100A5 ; 100B1 | 8 (49) |  |  |
|  | *P{PZ}zfh1^00865^* | 100A4 | 3 (86) |  |  |
|  | *PBac{5HPw+}dj-1β^A286^* | 100A5 | 8 (118) | 11 (259) |  |
|  | *wts^3-17^* | 100A5 | 0 (169) | 7 (113) |  |

^a^All Exelixis deficiencies from the primary screen that showed 10% malformed legs when heterozygous with *Rho1^E(br)246^*. ^b^Cytology is based upon flybase annotations as of January 2009 (reflects release 5 of the *Drosophila* genome). ^c^% malformed indicates the percentage of animals heterozygous for the indicated *Rho1* allele and heterozygous for the indicated deficiency or specific mutation showing the malformed leg phenotype in at least one leg. *n*, total number of flies of the indicated genotype that were scored. ^d^Data from (Ward RE, Evans J, Thummel CS 2003. Genetic modifier screens in *Drosophila* demonstrate a role for Rho1 signaling in ecdysone-triggered imaginal disc morphogenesis. Genetics 165: 1397-1415.)
